# Development of a salivary autoantibody biomarker panel for diagnosis of oral cavity squamous cell carcinoma

**DOI:** 10.3389/fonc.2022.968570

**Published:** 2022-10-31

**Authors:** Pei-Chun Hsueh, Kai-Ping Chang, Hao-Ping Liu, Wei-Fan Chiang, Xiu-Ya Chan, Chu-Mi Hung, Lichieh Julie Chu, Chih-Ching Wu

**Affiliations:** ^1^ Department of Fundamental Oncology, University of Lausanne, Lausanne, Switzerland; ^2^ Ludwig Institute for Cancer Research, University of Lausanne, Epalinges, Switzerland; ^3^ Department of Otolaryngology-Head and Neck Surgery, Linkou Chang Gung Memorial Hospital, Taoyuan, Taiwan; ^4^ Molecular Medicine Research Center, Chang Gung University, Taoyuan, Taiwan; ^5^ Department of Veterinary Medicine, College of Veterinary Medicine, National Chung Hsing University, Taichung, Taiwan; ^6^ Department of Oral and Maxillofacial Surgery, Chi-Mei Medical Center, Tainan, Taiwan; ^7^ Department of Medical Biotechnology and Laboratory Science, College of Medicine, Chang Gung University, Taoyuan, Taiwan; ^8^ Graduate Institute of Biomedical Sciences, College of Medicine, Chang Gung University, Taoyuan, Taiwan; ^9^ Research Center for Emerging Viral Infections, College of Medicine, Chang Gung University, Taoyuan, Taiwan

**Keywords:** autoantibody, saliva, oral cancer, biomarker, cancer screening

## Abstract

Oral cavity squamous cell carcinoma (OSCC) is a destructive disease with increasing incidence. OSCC is usually diagnosed at an advanced stage, which leads to poor outcomes of OSCC patients. Currently, there is a lack of biomarkers with sufficient effectiveness in early diagnosis of OSCC. To ameliorate OSCC screening, we evaluated the performances of salivary autoantibodies (auto-Abs) to nine proteins (ANXA2, CA2, ISG15, KNG1, MMP1, MMP3, PRDX2, SPARC, and HSPA5) as OSCC biomarkers. A multiplexed immunoassay using a fluorescence bead-based suspension array system was established for simultaneous assessment of the salivary levels of the above nine auto-Abs and a known OSCC-associated auto-Ab, anti-p53. Compared to healthy individuals (*n* = 140), the salivary levels of nine auto-Abs were significantly elevated in OSCC patients (*n* = 160). Notably, the salivary levels of the 10 auto-Abs in the early-stage OSCC patients (*n* = 102) were higher than that in the healthy group. Most importantly, utilizing a marker panel consisting of anti-MMP3, anti-PRDX2, anti-SPARC, and anti-HSPA5 for detection of early-stage OSCC achieved a sensitivity of 63.8% with a specificity of 90%. Collectively, herein we established a multiplex auto-Ab platform for OSCC screening, and demonstrated a four-auto-Ab panel which shows clinical applicability for early diagnosis of OSCC.

## Introduction

Oral cancer is one of health issues with global concerns and torments more than 500,000 individuals per year (1, 2). Oral cancer is prevalent and severe in Europe, South, and Central Asia (3, 4). In Taiwan, oral cancer is the fourth most common cancer among men and the fourth leading cause of cancer death (5, 6). Despite improved cancer elimination by advanced treatment approaches, the disease outcomes widely cause dysfunctions of chewing and swallowing and thus jeopardize patients’ life quality (7). Oral cavity squamous cell carcinoma (OSCC) accounts for more than 90% of all forms of oral cancers (8, 9) and exhibit high incidences of recurrence and cervical lymphatic metastasis (10). The majority of OSCC cases are diagnosed at advanced stages, which often associate with poor prognosis and treatment failure (10–12). Toward better clinical outcomes of OSCC, there is an urgent need to improve detection of OSCC at early stages.

Currently, OSCC diagnosis is mainly based on conventional oral examination (COE) followed by biopsy of suspected tissues for histopathological confirmation. Accurate and efficient detection of OSCC largely relies on experienced clinicians to visually inspect and palpate the presence of cancerous lesions of oral cavity. In this regard, development of an alternative detection approach combined with preliminary COE can improve OSCC diagnosis at early stages and benefit patients’ welfare (13–16). As OSCC cells are immersed in saliva, it is conceivable to detect OSCC-associated markers using saliva. Indeed, numerous efforts have been made to identify potential OSCC biomarkers in saliva using proteomics approaches (17–20). However, most of salivary biomarker candidates are neither saliva-accessible nor efficient enough for OSCC detection in practice due to a limit on sample size or sample quality for verification. As a result, there is a lack of biomarker-detection platforms that are readily accessible in clinics (21).

To develop a new approach for OSCC screening, we previously identified nine OSCC-associated proteins (ANXA2, CA2, ISG15, KNG1, MMP1, MMP3, PRDX2, SPARC, and HSPA5), and further determined that salivary levels of these nine proteins in OSCC patients are higher than that in healthy individuals (22). In respect with OSCC biomarkers, salivary autoantibodies (auto-Abs) against OSCC-associated proteins may be more clinically applicable compared with other molecules for their high specificity, stability, and abundance in saliva (23–26). In addition, reagents and platforms required for auto-Ab detection are well-established and readily accessible (27). Therefore, it is tempting to evaluate if the auto-Abs against the nine OSCC-associated proteins are detectable in salivary samples of OSCC patients and could be applied for OSCC detection.

In this study, we aim to evaluate the validity of utilizing salivary auto-Abs to the nine proteins for OSCC detection. To augment the detection effectiveness, a multiplexed bead-based system has been established to simultaneously detect levels of the nine auto-Abs in saliva samples collected from healthy individuals and OSCC patients. Our data showed that the salivary levels of auto-Abs (except anti-ANXA2) in the OSCC patients are higher than that in the healthy controls. Moreover, the salivary levels of all detected auto-Abs are elevated in patients with early-stage OSCCs compared with non-cancer individuals. Finally, a four-marker panel (anti-MMP3, anti-PRDX2, anti-SPARC, and anti-HSPA5) has been built to ameliorate the effectiveness of OSCC detection. Collectively, in this study, we established a multiplex auto-Ab detection platform and assembled an auto-Ab marker panel with clinical applicability for OSCC detection.

## Materials and methods

### Patient populations and clinical specimens

The salivary samples were collected from 140 healthy volunteers and 160 OSCC patients at the Chang Gung Memorial Hospital (CGMH), Linkou, Taoyuan, Taiwan from September 2010 to December 2018 and Chi-Mei Medical Center, Liouying, Tainan, Taiwan from January 2005 to August 2012 (**Table 1**). Salivary samples were collected followed the tenets of the Declaration of Helsinki. All volunteers permitted use of saliva specimens and signed an informed consent approved by the Institutional Review Board of the CGMH and Chi-Mei Medical Center before participation. All participants were examined by a routine oral mucosal screening test. Individuals with OSCC were proven through biopsy according to the standard protocol of oral cancer verification. Before collection of saliva samples, volunteers avoided eating, drinking, and smoking for at least 2 hours. Collected saliva samples were firstly centrifuged at 3,000 × g for 15 min at 4°C. The supernatants were immediately treated with a protease inhibitor mixture (2 μL/mL; Cat. No. P8340, Sigma-Aldrich, Burlington, MA, USA), then aliquoted into a volume of 100 μL, and stored at -80°C until use. To avoid protein degradation, saliva samples with more than one freeze-thaw cycle were not used.

**Table 1:** Characteristics of origins of saliva samples used for auto-Ab detection.

Characteristics	Control	OSCC
Case number	140	160^a^
No. of male/female	138/2	156/4
Median/range of age (years)	49/31-78	53/34-80
Cancer site		
Buccal mucosa	–	80
Gingiva	–	14
Lip	–	12
Tongue	–	36
Palate	–	10
Other	–	8^b^
No. of patients with well/moderate/poor differentiated cancers	–	98/54/8
No. of patients with T1/T2/T3/T4 pT status	–	62/58/20/20
No. of patients with N0/N1/N2 pN status	–	124/14/22
No. of patients with overall pathological stage-I/II/III/IV cancers	–	60/42/22/36

a There are 138, 6, and 16 patients with primary, second primary, and recurrent cancers, respectively.

b There are 2, 4, and 2 patients with cancers at the mouth floor, the retromolar trigone, and the neck, respectively.

### Reagents and antibodies

Six recombinant proteins were acquired from the R&D Systems (Minneapolis, MN, USA), including annexin A2 (ANXA2; Cat. No. 9409-AN-050), carbonic anhydrase 2 (CA2; Cat. No. 2184-CA-050), ubiquitin-like protein ISG15 (ISG15; Cat. No. UL-601-500), kininogen 1 (KNG1; Cat. No. 1569-PI-010), matrix metalloproteinase 1 (MMP1; Cat. No. 901-MP-010), and His-tagged secreted protein acidic and rich in cysteine (SPARC; Cat. No. 941-SP-050). His-tagged heat shock protein 70 family protein 5 (HSPA5; Cat. No. HSP-044) and His-tagged matrix metalloproteinase 3 (MMP3; Cat. No. ENZ-774) were purchased from the ProSpec (East Brunswick, NJ, USA). Human peroxiredoxin 2 (PRDX2) with His tag was bought from the Sino Biological (Cat. No. 11255-H07B, Beijing, China). Human p53 recombinant protein was in-house generated using *Escherichia coli* expression and purified as previously described (27). Anti-His Ab (Cat. No. AHP1656) and biotin-conjugated goat anti-human IgA (Cat. No. 205008) were acquired from the Bio-Rad Laboratories (Hercules, CA, USA). Phycoerythrin-labeled streptavidin (SA-PE) was obtained from the Jackson ImmunoResearch Laboratories (Cat. No. 016-110-084, West Grove, PA, USA). N-hydroxysulfosuccinimide (NHS; Cat. No. 24500) and 1-ethyl-3-(3-dimethylaminopropyl) carbodiimide (EDC; Cat. No. 22980) were purchased from the Thermo Fisher Scientific (Waltham, MA, USA).

### Establishment of a bead-based immunoassay for auto-Ab detection

The recombinant proteins were covalently coupled to carboxylated polystyrene beads following standard protocol of Bio-Plex amine coupling kit (Cat. No. 171406001, Bio-Rad Laboratories, Hercules, CA, USA). Briefly, 1.5 × 10^6^ Bio-Plex COOH beads were activated with 80 μL activation buffer (2-(*N*-morpholino)ethanesulfonic acid, 0.1 M, pH 5.4). Then 10 μL activation buffer with NHS (50 mg/mL) and EDC (50 mg/mL) were added. After 20 min at room temperature, the beads were washed twice with 150 μL phosphate buffered saline (PBS; pH 7.4) and incubated with the recombinant proteins for 2 hours at room temperature. Six μg of the proteins were used while 12 μg for MMP3 and PRDX2 proteins. Finally, the beads were washed with 500 μL PBS (pH 7.4), blocked with 250 μL blocking buffer, resuspended and stored in 500 μL storage buffer at 4°C until use. Coupling efficiency of each protein was verified with anti-His (Bio-Rad Laboratories) or its specific antibody using the Bio-Plex 200 system (Cat. No. 171000205, Bio-Rad Laboratories).

### Multiplexed detection of salivary IgA autoantibodies

The assays were conducted with 96-well filter-bottom microplates (Cat. No. MSBVN1B50, Merck Millipore, Taipei, Taiwan) in a dark room to avoid light. The recombinant protein-conjugated beads (5000 beads for each protein) were firstly mixed and washed in microplates. Each saliva sample was diluted by a factor of 25 with PBS containing 1% BSA (Cat. No. A2153, Sigma-Aldrich), added into the microplates, and incubated for 1 hour at room temperature. After washing, 50 μL of biotin-conjugated anti-human IgA (1 μg/mL) in PBS containing 1% BSA was applied. After incubation for 40 minutes, 50 μL of SA-PE (1000-fold dilutions) in PBS containing 1% BSA was added. After incubation of 20 minutes, fluorescence intensities of bead identities and SA-PE were detected using Bio-Plex 200 system and the Bio-Plex Manager software version 4.2 (Bio-Rad Laboratories).

### Statistical analysis

Levene’s test was used to determine whether variances of two groups are equal. Comparison of salivary auto-Ab levels between groups was evaluated by a two-sample t-test. The 90^th^ percentile of median fluorescence intensities (MFI) in the control group was set as cut-off value to obtain sensitivity and specificity of each auto-Ab in the other groups. Receiver operating characteristic (ROC) curve was used to evaluate capability of auto-Abs for discriminating OSCC patients from healthy individuals. All data were performed using the SPSS software (version 20, IBM, Armonk, NY, USA). A *p* value < 0.05 was considered statistically significant.

## Results

### Establishment of a multiplexed immunoassay for profiling of salivary auto-Abs

**Figure 1:**
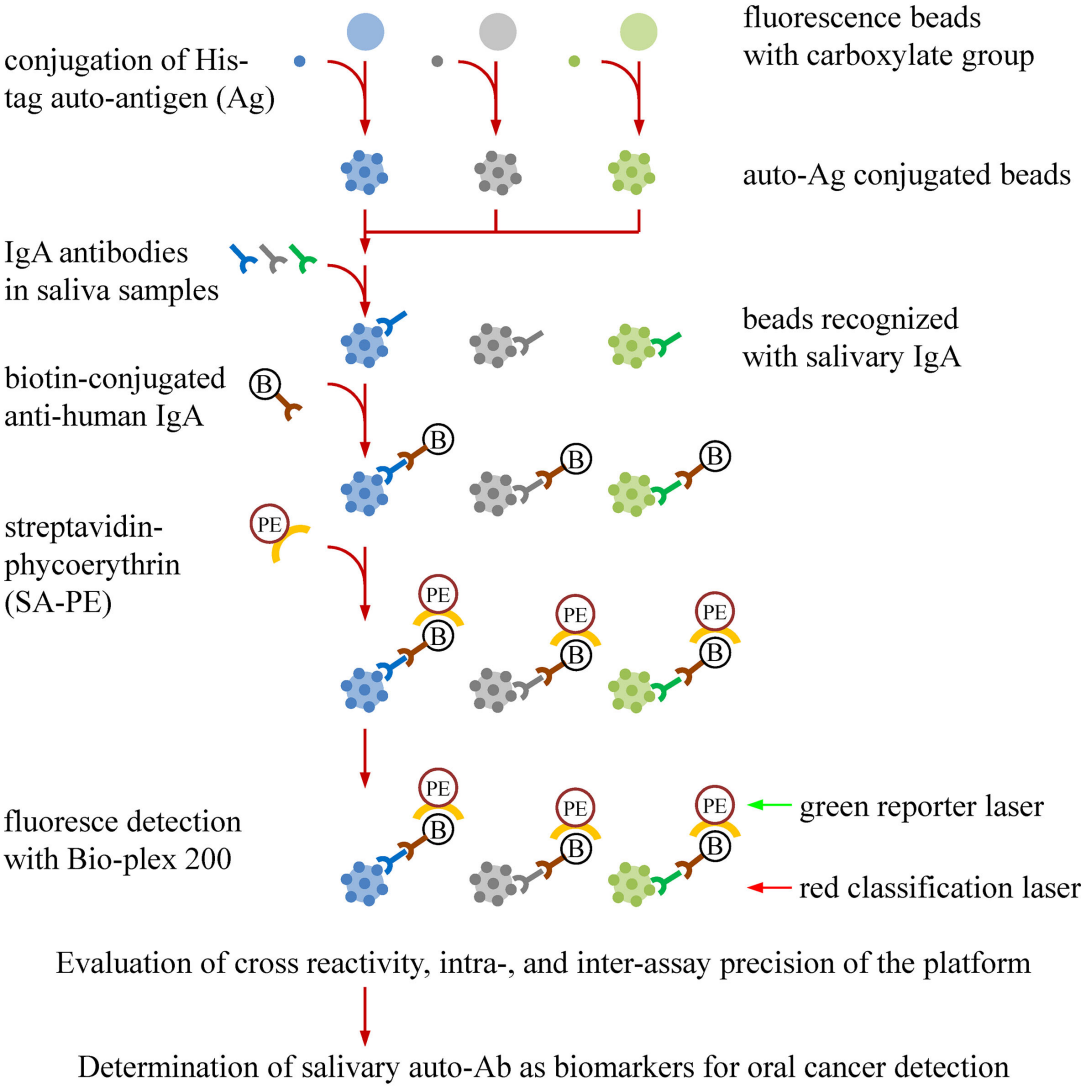
Workflow for establishment of a bead-based suspension immunoassay for auto-Ab detection in saliva samples. Auto-Ab detection was conducted with a multiplexed bead-based suspension array system (Bio-Plex). To generate the beads used in the system, individual His-tagged recombinant protein were covalently conjugated to the COOH beads with an unique fluorescent identity. The resulting recombinant protein-conjugated beads can capture the salivary IgA specifically responsive to the recombinant proteins. By addition of a biotin-labeled anti-human IgA and streptavidin-phycoerythrin (SA-PE), the levels and identities of salivary IgA auto-Abs can be simultaneously investigated in the Bio-Plex system, in which the “red” laser is applied to interrogate the bead identity which identifies the auto-Ab type, and the “green” laser is used to assess the level of the identified auto-Abs. An anti-His Ab is used to evaluate the protein-coupling efficiency, the cross-reactivity of the beads, and the precision of the multiplex assay.

We previously identified nine OSCC-related proteins (ANXA2, CA2, ISG15, KNG1, MMP1, MMP3, PRDX2, SPARC, and HSPA5) with elevated levels in saliva of OSCC patients by multiple-reaction-monitoring mass spectrometry (22). To evaluate the validity of utilizing the nine auto-Abs in saliva for OSCC detection, we established a multiplexed auto-Ab assay by using a fluorescence bead-based suspension array system to simultaneously survey salivary levels of the nine auto-Abs and anti-p53, which is well-known for its potential usefulness in OSCC detection (**Figure 1**).

**Figure 2:**
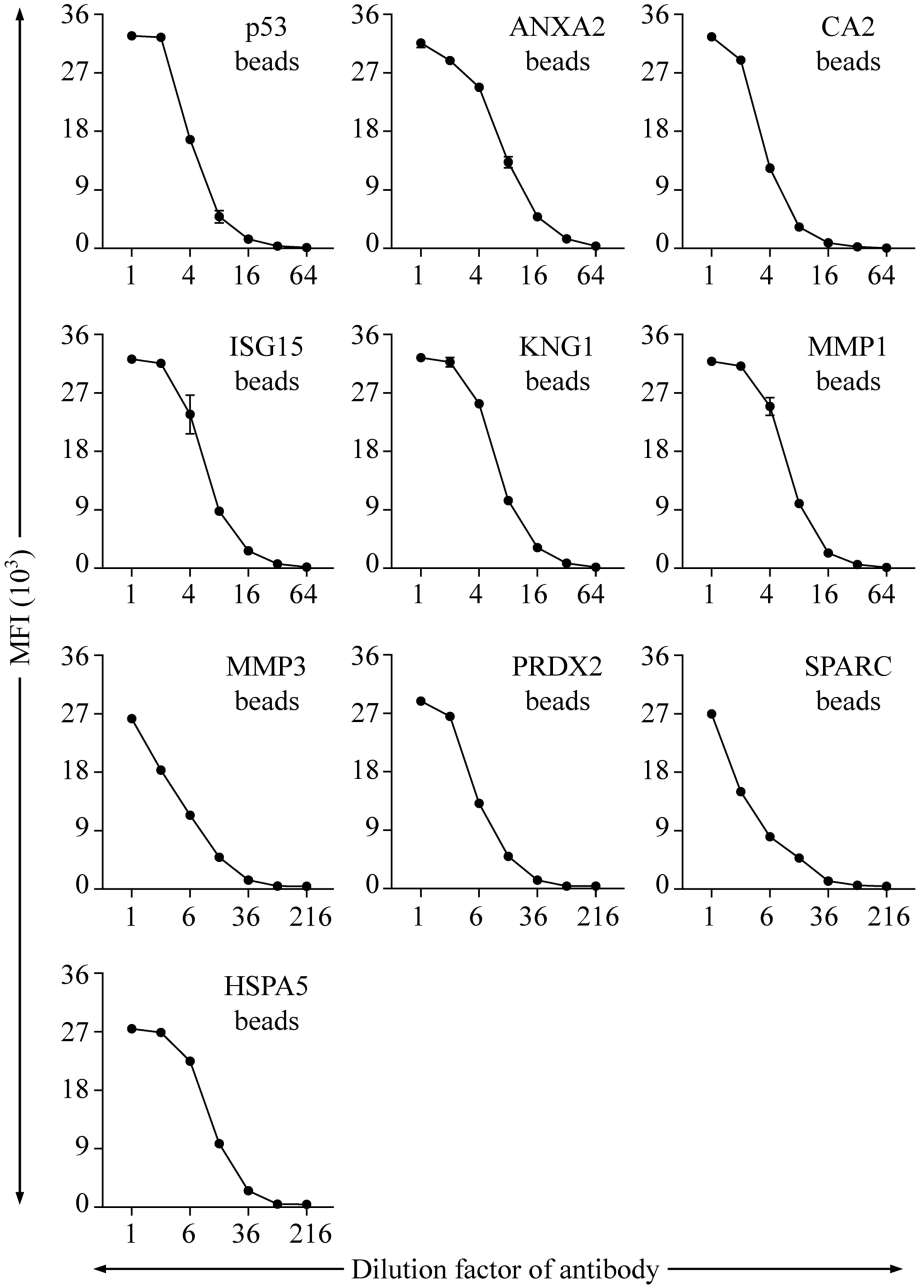
Evaluation of the range and efficiency of auto-Ab detection with the established bead-based suspension immunoassay. The protein-coupling efficiency of individual beads and their effectiveness in detection of corresponding auto-Abs were verified by using Abs specific to individual proteins, including p53, ANXA2, CA2, ISG15, KNG1, and MMP1, respectively. An anti-His Ab was applied for verification of the beads coupled with His-tagged MMP3, PRDX2, SPARC, and HSPA5, respectively. Initial concentrations of the Abs specific to p53, ANXA2, CA2, ISG15, KNG1, MMP1, and His tag are 0.03, 4, 0.2, 0.2, 20, 0.4, and 20 μg/mL, respectively. Data are acquired as the median fluorescence intensity (MFI) and shown as the mean ± SD of MFI.

To evaluate the protein-labeling efficiency and antibody-recognition ability of the protein-conjugated beads, specific Abs to six proteins (p53, ANXA2, CA2, ISG15, KNG1, and MMP1), respectively, were used to verify the corresponding protein-conjugated beads. On the other hand, the beads linked with MMP3, PRDX2, SPARC, and HSPA5 were individually detected with an anti-His Ab. As shown in **Figure 2**, each of the specific Abs can be efficiently and dose-dependently detected with the corresponding beads in the fluorescence bead-based suspension array system.

### Performance assessment of the multiplexed immunoassay for salivary auto-Ab detection

**Figure 3:**
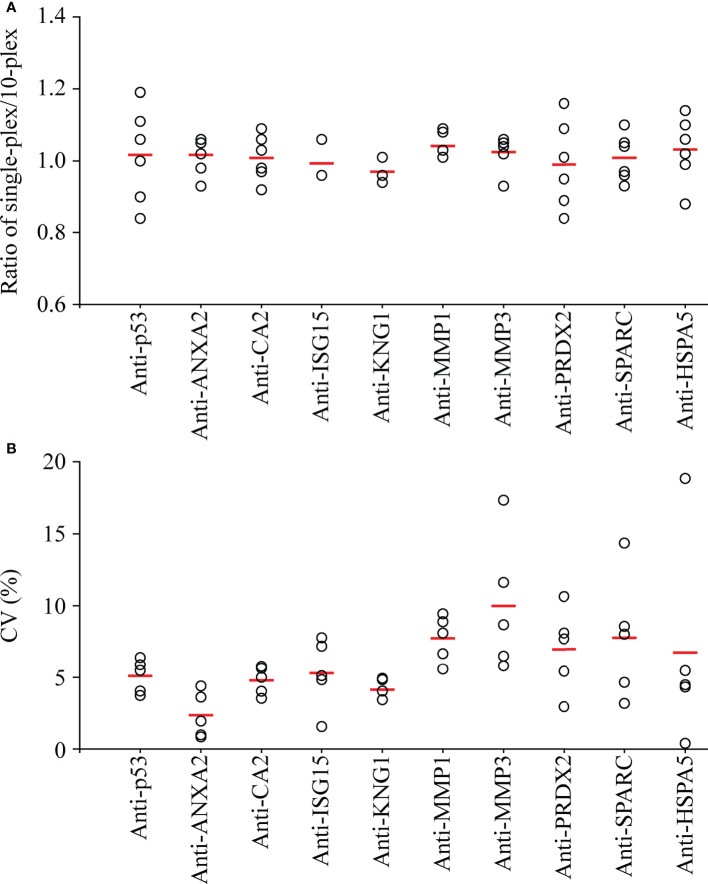
Assessment of the cross-reactivity and inter-assay precision of the multiplexed auto-Ab immunoassay. **(A)** To evaluate the cross-reactivity of the multiplexed immunoassay, six pooled salivary samples, each of which was pooled from saliva samples of six OSCC patients and six healthy controls, were subjected to detection of the 10 auto-Abs using individual protein-conjugated beads (single-plex) and using equally mixed beads respectively for 10 auto-Abs (10-plex) in parallel. Results are presented as a ratio of the auto-Ab level acquired in single-plex to that in 10-plex for each of the six pooled samples (open circles), and the mean auto-Ab ratio of the six pooled samples is indicated with a red thick line. **(B)** To investigate the inter-assay precision of the 10-plex immunoassay, measures of auto-Ab levels in five pooled saliva samples were taken at three different time points. Results are presented as the coefficient of variation (CV) of auto-Ab levels acquired from the three batches of individual pooled samples (open circles), and the mean CV of the auto-Ab in the five samples is indicated with a red thick line.

For setting of a 10-plex auto-Ab immunoassay, the 10 types of protein-conjugated beads were mixed in equal quantities. To evaluate the cross-reactivity in this 10-plex assay, six saliva samples pooled from 12 individual saliva specimens were subjected to assessment of the salivary level of individual auto-Ab using a 10-plex setting compared with that using a single-target assay (**Supplementary Figure S1**). As shown in **Figure 3A**, the ratio of the auto-Ab level determined with a single-target assay to that determined with a 10-plex setting was close to one (ranged between 0.84 and 1.19) in each case, revealing that the 10-plex assay achieves multiplexed detection of salivary auto-Abs with a limited cross-reactivity.

To evaluate the reliability of salivary auto-Ab detection with the 10-plex immunoassay, the 10 auto-Abs were detected in the pooled saliva samples in five replicates in a same run to access the intra-assay precision. As shown in **Supplementary Figure S2**, the mean of the coefficient of variation (mean CV) of intra-assays ranged from 5.60% to 8.79%. Moreover, measure of individual auto-Ab levels in the five replicated samples were taken three times at different time points to evaluate the inter-assay precision. The mean CVs of the inter-assays were lower than 10% (2.51%-9.12%) in detection of each auto-Ab (**Figure 3B**). Data reveal that this 10-plex assay is accurate and efficient for detection of the auto-Abs in saliva samples.

### Profiling of the 10 auto-Abs in saliva samples of OSCC patients

**Figure 4:**
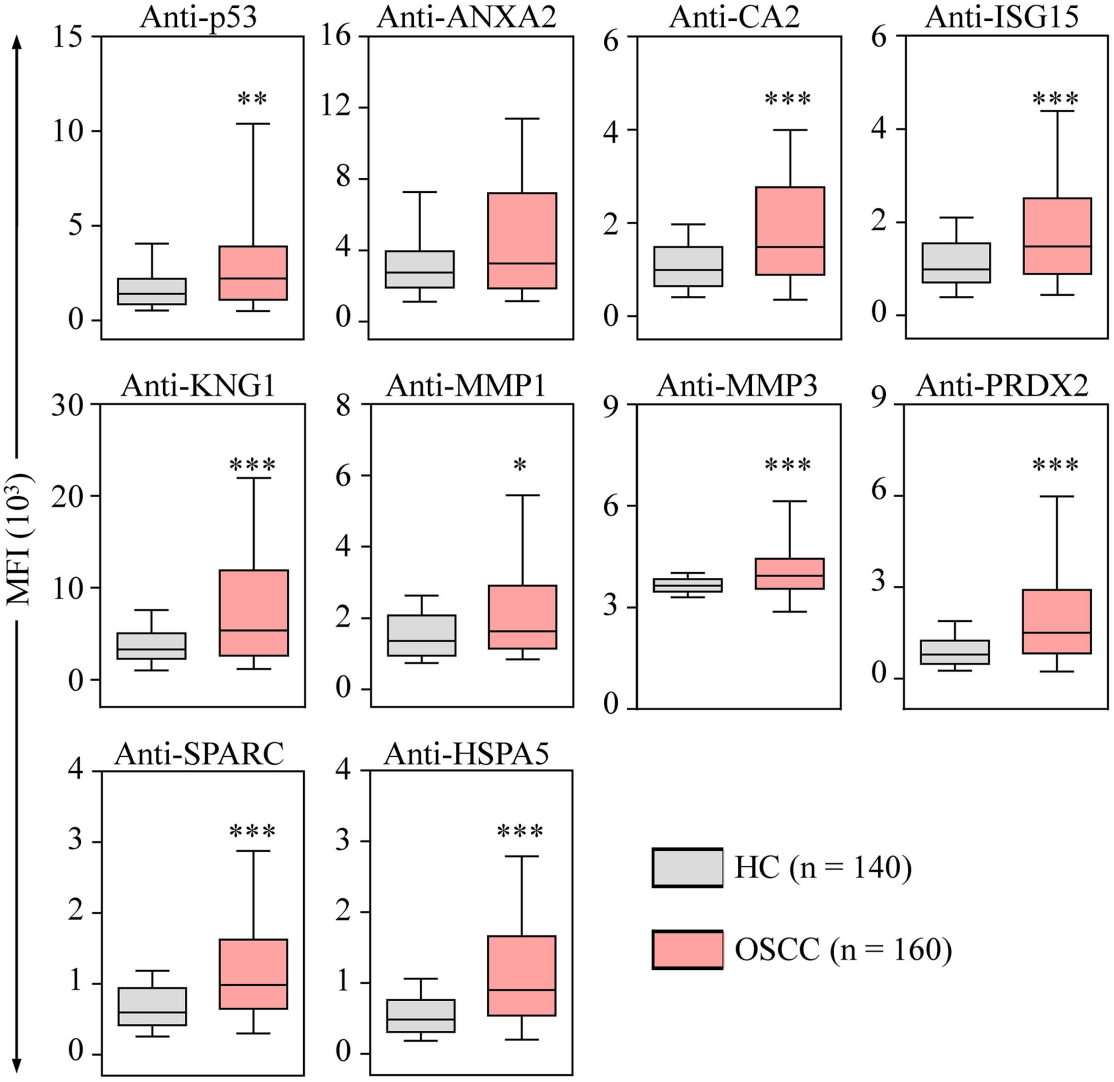
Elevated levels of salivary auto-Abs in the OSCC patients. The levels of auto-Abs were detected in saliva samples respectively collected from healthy controls (HC; *n* = 70), and OSCC patients (*n* = 80) with the multiplexed bead-based system. Salivary levels of the auto-Abs are shown with the median fluorescence intensity (MFI). Data are presented as the upper and lower quartiles (box), the median value (the horizontal line), and the middle 90% distribution (the whisker) of MFI. *p < 0.05, **p < 0.01 and ***p < 0.001.

To evaluate the performance of utilizing the 10 auto-Abs as salivary OSCC biomarkers, salivary levels of the 10 auto-Abs were assessed in saliva samples from 140 healthy individuals and 160 OSCC patients using the 10-plex assay (**Table 1** and **Supplementary Table S1**). As shown in **Figure 4**, the salivary levels of all the auto-Abs in the OSCC patients were higher than that in the healthy group, although it is not statistically significant enough in the case of anti-ANXA2. Of note, the salivary levels of eight auto-Abs in the patients with primary OSCC were significantly elevated compared with those in the healthy controls (**Table 2**), suggesting potentials of these auto-Abs (anti-p53, anti-CA2, anti-ISG15, anti-KNG1, anti-MMP3, anti-PRDX2, anti-SPARC, and anti-HSPA5) as salivary biomarkers for OSCC screening. Moreover, the salivary levels of the 10 auto-Ab were all elevated in the OSCC-recurrent patients. On the other hand, the salivary levels of anti-ANXA2, anti-KNG1, and anti-MMP1 in the relapsed OSCC group were much higher than that in the primary OSCC group (**Table 2**). However, it is necessary to expand the case number of relapsed OSCC for further validation of these 3 auto-Abs as biomarkers of OSCC recurrence.

**Table 2:** Salivary levels of auto-antibodies (auto-Abs) in OSCC patients.

Auto-Abs	Median fluorescence intensity (MFI) of auto-Abs^a^								
	Healthycontrols (*n* = 140)^a^	OSCC patients							
		PrimaryOSCC (*n* = 135)	*p*value^b^	RecurrentOSCC (*n* = 25)	*p*value^b^	Overallpathologicalstage I-II (*n* = 102)	*p*value^b^	Overallpathologicalstage III-IV(*n* = 58)	*p*value^b^
Anti-p53	1771 ± 1293	3388 ± 4277	<0.05	5923 ± 5501	<0.05	3973 ± 4775	<0.01	3059 ± 3785	0.181
Anti-ANXA2	3361 ± 2661	4514 ± 4278	0.170	8182 ± 6176	<0.05	5607 ± 5179	<0.05	3604 ± 2974	0.887
Anti-CA2	1101 ± 653	2203 ± 2943	<0.01	2756 ± 1935	<0.01	2536 ± 3403	<0.001	1769 ± 1385	<0.05
Anti-ISG15	1162 ± 679	2268 ± 3156	<0.01	2934 ± 2189	<0.05	2637 ± 3681	<0.001	1803 ± 1379	0.062
Anti-KNG1	4043 ± 2984	7865 ± 7966	<0.01	13336 ± 9104	<0.01	8526 ± 8282	<0.001	8213 ± 8171	<0.05
Anti-MMP1	1591 ± 967	2510 ± 3054	0.057	4474 ± 3150	<0.01	2926 ± 3531	<0.05	2319 ± 2150	0.143
Anti-MMP3	3660 ± 1377	4398 ± 2414	<0.001	4496 ± 1710	<0.05	4516 ± 2593	<0.01	4216 ± 1855	<0.05
Anti-PRDX2	1025 ± 928	2353 ± 2948	<0.001	2912 ± 1903	<0.01	2619 ± 3058	<0.001	2041 ± 2470	<0.05
Anti-SPARC	639 ± 385	1622 ± 2877	<0.001	1904 ± 1260	<0.01	1953 ± 3379	<0.001	1118 ± 748	<0.01
Anti-HSPA5	568 ± 354	1406 ± 2270	<0.001	1961 ± 1711	<0.01	1718 ± 2697	<0.001	1011 ± 752	<0.01

a MFI for the indicated group was shown as mean ± standard deviation (SD).

b The p values for the comparison between the indicated group and the healthy control group were determined by a two-sample t-test.

### Effectiveness of utilizing salivary auto-Abs for OSCC detection

To examine the performance of the auto-Abs as salivary OSCC biomarkers, receiver operating characteristic (ROC) curve analysis was employed to evaluate the effectiveness of using the auto-Abs in discriminating the OSCC patients from the healthy individuals. As shown in **Supplementary Table S2**, the area under ROC curve (AUC) values of the auto-Abs to CA2, ISG15, KNG1, MMP3, PRDX2, SPARC, and HSPA5, respectively, were greater than 0.66, in particular the AUC values of anti-SPARC and anti-HSPA5 were 0.712 (95% CI: 0.629-0.795) and 0.717 (95% CI: 0.634-0.800), respectively (**Supplementary Table S2**). Diagnostic accuracy of the auto-Abs was further determined by calculating their sensitivities (true positive rates). With a cut-off point of 90% specificity (true negative rate), the sensitivity of anti-p53 as an OSCC screening biomarker was 25.0%. Sensitivities of the other nine auto-Abs ranged between 28.8% and 46.3% (**Table 3**), all of which were superior to that of anti-p53. Notably, the sensitivities of anti-MMP3, anti-PRDX2, anti-SPARC, and anti-HSPA5 were more than 40%. Most importantly, using a marker panel composed of these four auto-Abs greatly improved the sensitivity of OSCC diagnosis (63.8%) compared with using the individual marker alone (**Table 3**). For distinguishing the OSCC patients from the healthy controls, the AUC value of using the four-auto-Ab panel was greater than that of using either marker alone (AUC = 0.806; 95% CI: 0.758-0.854; **Supplementary Figure S3**), suggesting that this four-marker panel was applicable for screening OSCC with saliva samples.

**Table 3:** Sensitivities of salivary auto-Ab biomarkers for OSCC detection.

Auto-Abs	Cut-off valueof MFI^a^	No./percentage of auto-Ab positive cases			
		Healthycontrol (*n* = 140)	OSCC(*n* = 160)	Overallpathologicalstage I-II (*n* = 102)	Well-differentiatedOSCC (*n* = 98)
Anti-p53	3942.2	14/10.0%	40/25.0%	28/27.5%	32/32.7%
Anti-ANXA2	5994.4	14/10.0%	46/28.8%	34/33.3%	30/30.6%
Anti-CA2	1951.5	14/10.0%	58/36.3%	40/39.2%	40/40.8%
Anti-ISG15	1059.9	14/10.0%	52/32.5%	36/35.3%	36/36.7%
Anti-KNG1	2078.6	14/10.0%	58/36.3%	34/33.3%	40/40.8%
Anti-MMP1	7379.6	14/10.0%	54/33.8%	38/37.3%	38/38.8%
Anti-MMP3	2555.5	14/10.0%	74/46.3%	46/49.0%	50/51.0%
Anti-PRDX2	3997.3	14/10.0%	66/41.3%	48/47.1%	50/51.0%
Anti-SPARC	1845.0	14/10.0%	74/46.3%	52/51.0%	42/42.9%
Anti-HSPA5	1176.7	14/10.0%	68/42.5%	48/47.1%	44/44.9%
Four-marker panel^b^	either 1 positive	14/10.0%	102/63.8%	72/70.6%	70/71.4%

a With a given specificity of 90%, the MFI of each auto-Ab is set as the cutoff value.

b The panel is consisted of auto-Abs respectively responsive to MMP3, PRDX2, SPARC, and HSPA5.

### Performance of salivary auto-Abs in detection of early-stage OSCC

To assess the performance of the auto-Abs in detection of early-stage OSCC, we investigated the salivary auto-Ab levels in patients with OSCC at different clinical stages or statuses according to overall pathological stage, primary tumor size (TNM-T classification), lymphatic metastasis (TNM-N classification), and cell differentiation. Compared with the healthy individuals, salivary levels of seven auto-Abs (Abs to CA2, ISG15, KNG1, MMP3, PRDX2, SPARC, and HSPA5, respectively) and anti-p53 were significantly elevated in the patients with OSCC at overall pathological stage I-II (**Table 2** and **Supplementary Figure S4**), at TNM-T1 (**Supplementary Figure S5**), at TNM-N0 (**Supplementary Figure S6**), and those with well-differentiated OSCC (**Supplementary Figure S7**). Notably, the salivary levels of anti-p53, anti-ANXA2, anti-ISG15, and anti-MMP1 in the overall pathological stage I-II and the TNM-N0 cancer groups were higher than that in the control groups, while the salivary levels of the above four auto-Abs were not significantly distinguishable between the groups of late-stage OSCC patients and the healthy individuals (**Table 2**, **Supplementary Figures S4** and S6).

Furthermore, the AUC values of utilizing the salivary auto-Abs in detection of early-stage OSCC (stage I-II) were analyzed. For distinguishing the patients with OSCC at overall pathological stage I-II from the healthy controls, the AUC values of anti-CA2, anti-ISG15, anti-KNG1, anti-MMP3, anti-PRDX2, anti-SPARC, and anti-HSPA5 were greater than that of anti-p53 (AUC = 0.647; 95% CI: 0.544-0.751). Notably, the AUC values of anti-PRDX2, anti-SPARC, and anti-HSPA5 were higher than 0.72 (**Supplementary Table S2**). The sensitivities of utilizing the auto-Abs for detecting early-stage OSCC were further determined. Given a specificity of 90%, utilizing anti-p53 had a sensitivity of 27.5% for identification of OSCC at overall pathological stage I-II. The sensitivities of utilizing the other auto-Abs ranged from 33.3% to 51.0%, superior to that of utilizing anti-p53. More importantly, using the four-marker panel can detect 72 out of 102 (70.6%) patients with early-stage OSCC (**Table 3**), which indicates that the marker panel consisting of anti-MMP3, anti-PRDX2, anti-SPARC, and anti-HSPA5 is potentially practicable in early diagnosis of OSCC.

### Correlations between salivary levels of auto-Abs and OSCC characteristics

The relationship between clinical manifestations of OSCC and the salivary auto-Ab levels was then inspected. As shown in **Supplementary Table S3**, higher levels of salivary anti-p53, anti-CA2, anti-ISG15, and anti-SPARC were significantly associated with habitual behaviors of chewing betel nut and alcohol consumption. The salivary levels of anti-KNG1 and anti-PRDX2 in betel quid chewers were much higher than that in individuals without the customary behavior. Compared with the individuals without habitual behavior of drinking alcohol, the individuals with alcohol consumption habits had an elevated level of anti-HSPA5 (**Supplementary Table S3**). In addition, the salivary anti-PRDX2 level was increased in the patients with OSCC at TNM-N0 stage compared with that in the patients with OSCC at TNM-N > 0 stage. However, analysis with an expanded number of OSCC patients with lymphatic metastasis is needed to confirm this significance (**Supplementary Table S3**).

Despite a lack of statistical significance, the salivary auto-Ab levels appeared to decline in the late-stage OSCC patients compared with those in the early-stage OSCC patients (**Table 2** and **Supplementary Figure S4**). Similar phenomena were also observed in the comparisons between the groups of OSCC with and without lymphatic metastasis (**Supplementary Table S3** and **Figure S6**), and in the comparison between the groups of the moderate-differentiated OSCC and the well-differentiated cancers (**Supplementary Table S3** and **Supplementary Figure S7**). Moreover, the salivary auto-Ab levels were not correlated with the age and smoking of patients, differentiation status of cancer cell, TNM-T classification, or cancer site in the present case-control analysis.

## Discussion

As a global health burden, oral cancer is prevalent and severe in south and central Asia (1, 2). Early diagnosis of OSCC can significantly improves outcomes after treatment. Conducting traditional COE combined with detection of relevant biomarkers can achieve better detection of OSCC at early stages (28). To this end, in the present study, we established a multiplexed bead-based immunoassay to simultaneously detect salivary auto-Abs to p53, ANXA2, CA2, ISG15, KNG1, MMP1, MMP3, PRDX2, SPARC, and HSPA5, respectively (**Figure 1**). Compared with the single auto-Ab assay, the established multiplex auto-Ab assay provides robust detection of multiple auto-Abs with high sensitivity and specificity yet reduced sample usage and lower costs, and could be clinically applicable for a survey of a marker panel of OSCC.

In this study, we demonstrated that the salivary levels of anti-Abs to p53, CA2, ISG15, KNG1, MMP1, MMP3, PRDX2, SPARC, and HSPA5, respectively, are significantly higher in the OSCC patients compared to those in the healthy individuals (**Table 2** and **Figure 4**). More importantly, the levels of 10 auto-Abs are also elevated in the early-stage OSCC group (**Table 2** and **Supplementary Figure S4**). Among the 10 auto-Abs, anti-p53 is a well-characterized Ab biomarker of OSCC (27), which reaches a sensitivity of 25.0% with a specificity of 90.0% for OSCC detection (**Table 3**). Notably, the other nine auto-Abs analyzed in this study achieve a sensitivity of 28.5% and above for OSCC detection (**Table 3**), suggesting that the nine auto-Abs may hold greater potentials of serving as screening biomarkers of OSCC. Despite the insufficient sensitivity of using individual auto-Ab for OSCC detection, utilizing the four-marker panel composed of anti-MMP3, anti-PRDX2, anti-SPARC, and anti-HSPA5 has been shown to be much more sensitive in OSCC detection, and is especially useful for identification of early-stage OSCC (sensitivity: 63.8%; specificity: 90%) and well-differentiated OSCC (sensitivity: 71.4%; specificity: 90.0%; **Table 3**).

In the relapsed OSCC group, the salivary levels of anti-ANXA2, anti-KNG1, and anti-MMP1 are higher than those in the primary OSCC group, in particular the salivary anti-MMP1 level, which is most significantly elevated in the patients with recurrent OSCC (MFI: 4474 ± 3150) compared to the level in those with primary OSCC (MFI: 2510 ± 3054; *p* = 0.028; **Table 2**). It is worthy to confirm this significance using a cohort of larger sample size. It has been suggested that cancer immunosurveillance during tumorigenesis may lead to increased production of cancer-associated auto-Abs (29–31) in virtue of developing immunogenicity or loss of self-tolerance to self-antigens (32, 33). On the other hand, the salivary auto-Ab levels in the patients with advanced-stage OSCC at overall pathological stage III-IV and at TNM-N > 0 status are relatively lower than those in the patients with OSCC at early stage and at TNM-N0 status, respectively (**Table 2**, **Supplementary Table S3**, **Supplementary Figures S4** and S6). Such outcomes may arise from evasions of the late-stage OSCC cells from immunosurveillance through generation of poorly immunogenic cancer cells and through subversion of the immune system (34, 35).

In sum, herein we established a multiplexed auto-Ab immunoassay for OSCC detection which shows a value of clinical applicability. Moreover, we developed a four-auto-Ab panel which is effective in detection of early-stage OSCC. Detecting the auto-Ab panel paired up with traditional COE could greatly ameliorate the effectiveness of OSCC detection, and thereby enable patients to receive tailored treatment regimens to achieve better disease outcomes.

## Data availability statement

The raw data supporting the conclusions of this article will be made available by the authors, without undue reservation.

## Ethics statement

The studies involving human participants were reviewed and approved by the Institutional Review Board at Chi-Mei Medical Center, Taiwan (IRB No. 10012-L02) and Chang Gung Memorial Hospital, Taiwan (IRB No. 201800700B0 and 102-5685A3). Prior to sample collection, written informed consent was obtained from all participants. The patients/participants provided their written informed consent to participate in this study.

## Author contributions

Conceptualization, P-CH and C-CW. Formal analysis, P-CH, H-PL and C-MH. Investigation, K-PC and C-CW. Resources, K-PC and LC. Supervision, C-CW. Writing-original draft, P-CH, K-PC and C-CW. Review and editing of writing, H-PL and C-CW. Methodology, X-YC, C-MH and LC. All authors contributed to the article and approved the submitted version.

## Funding

This research was supported by grants to C-CW from the Ministry of Science and Technology, Taiwan (108-2320-B-182-030-MY3), and the Chang Gung Memorial Hospital (BMRPC77). This work was also financially supported by the Research Center for Emerging Viral Infections from the Featured Areas Research Center Program within the framework of the Higher Education Sprout Project by the Ministry of Education (MOE), Taiwan, and the Ministry of Science and Technology, Taiwan (110-2634-F-182-001).

## Acknowledgments

We acknowledge the Proteomics Core Laboratory at Chang Gung University, Taoyuan, Taiwan, for technical support (CLRPD1J0013).

## Conflict of interest

The authors declare that the research was conducted in the absence of any commercial or financial relationships that could be construed as a potential conflict of interest.

## Publisher’s note

All claims expressed in this article are solely those of the authors and do not necessarily represent those of their affiliated organizations, or those of the publisher, the editors and the reviewers. Any product that may be evaluated in this article, or claim that may be made by its manufacturer, is not guaranteed or endorsed by the publisher.
