# Supplementary material for: Development of a salivary autoantibody biomarker panel for diagnosis of oral cavity squamous cell carcinoma
Source: Front Oncol. 2022 Oct 31;12:968570. doi: 10.3389/fonc.2022.968570 (PMC9659860; doi:10.3389/fonc.2022.968570)
Supplement: Supplementary file 1 [file DataSheet_1.pdf]

Supplementary Figure S1

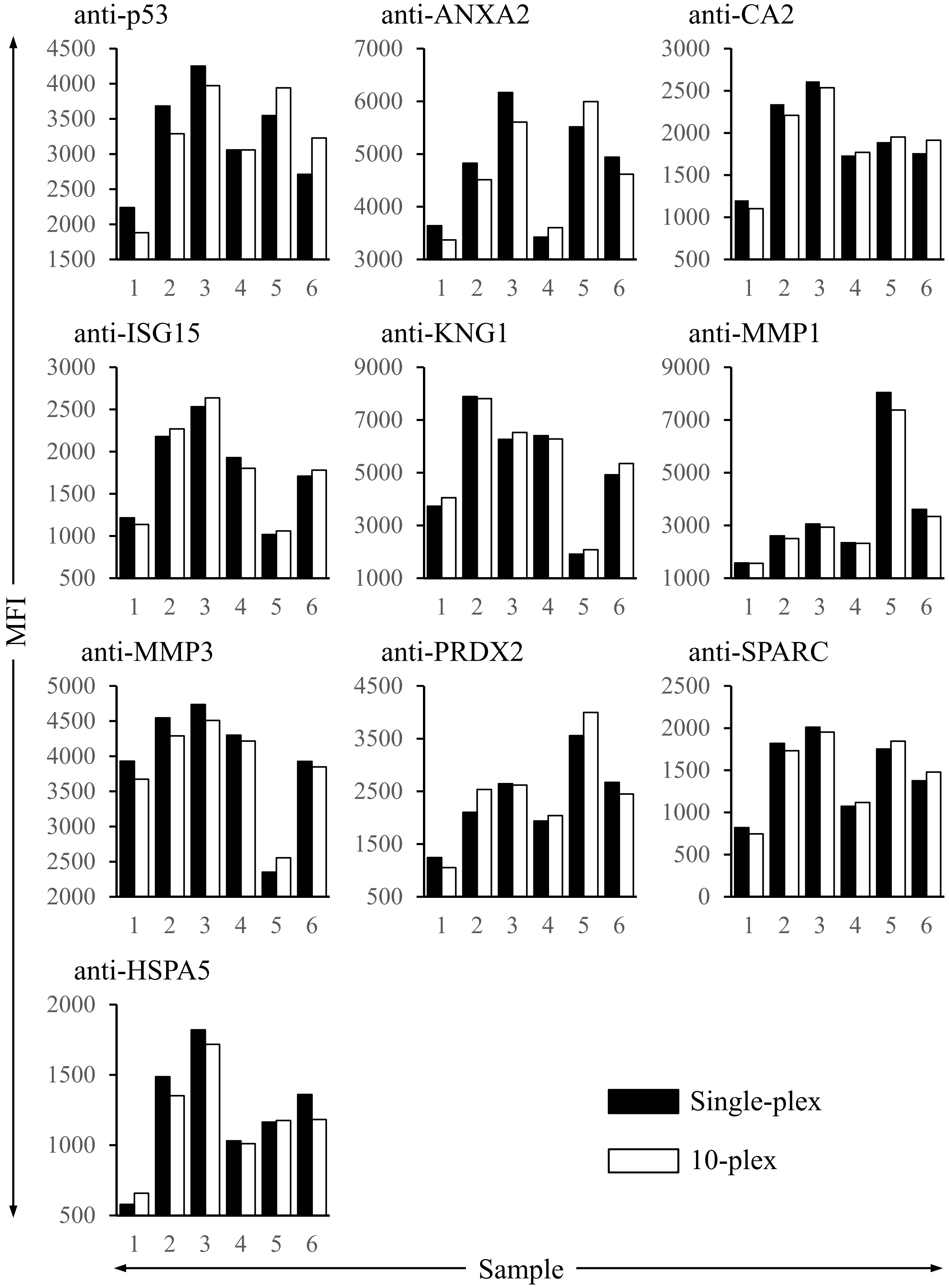

Supplementary Figure S1. Assessment of cross-reactivity of the multiplexed auto-Ab assay. To evaluate cross-reactivity of the auto-Ab immunoassay, level of auto-Ab in 6 pooled salivary samples (each was pooled from 12 saliva samples) was determined both with the individual beads (single-plex) and equally-mixed beads (10-plex). Results are shown as median fluorescence intensity (MFI) of the auto-Ab acquired in single-plex (closed bars) to that in 10-plex (open bars) for each samples.

Supplementary Figure S2

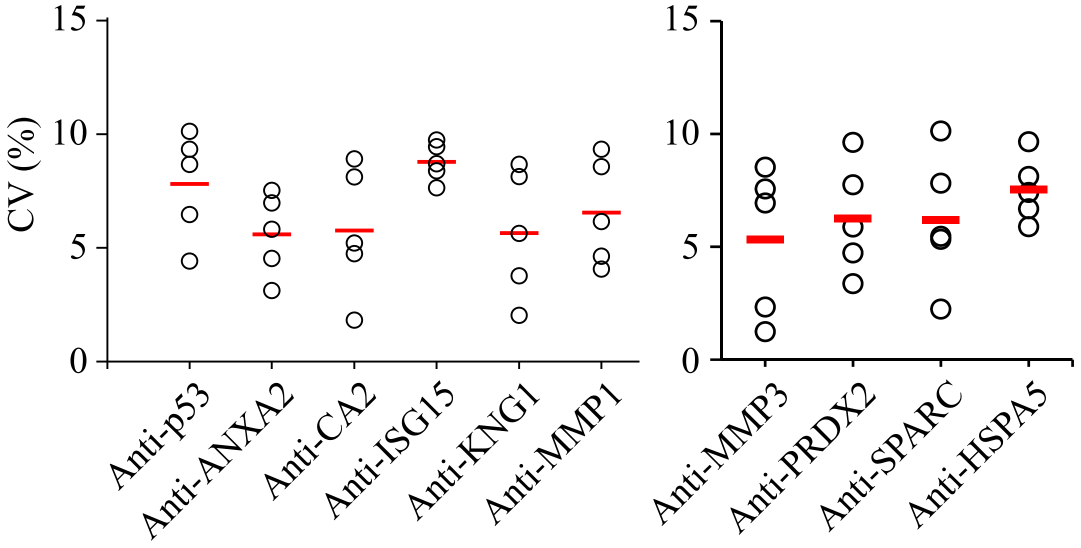

Supplemental Figure S2. Evaluation of intra-assay precision of the multiplexed auto-Ab detection platform. The levels of auto-Abs were detected in five saliva samples for five replicates at the same time to access the intra-assay precision of the established bead-based immunoassay. Data is presented as the coefficient of variation (CV) of five replicates for each salivary sample (open circles) and the mean of CVs for the five samples is shown with red thick lines.

Supplementary Figure S3

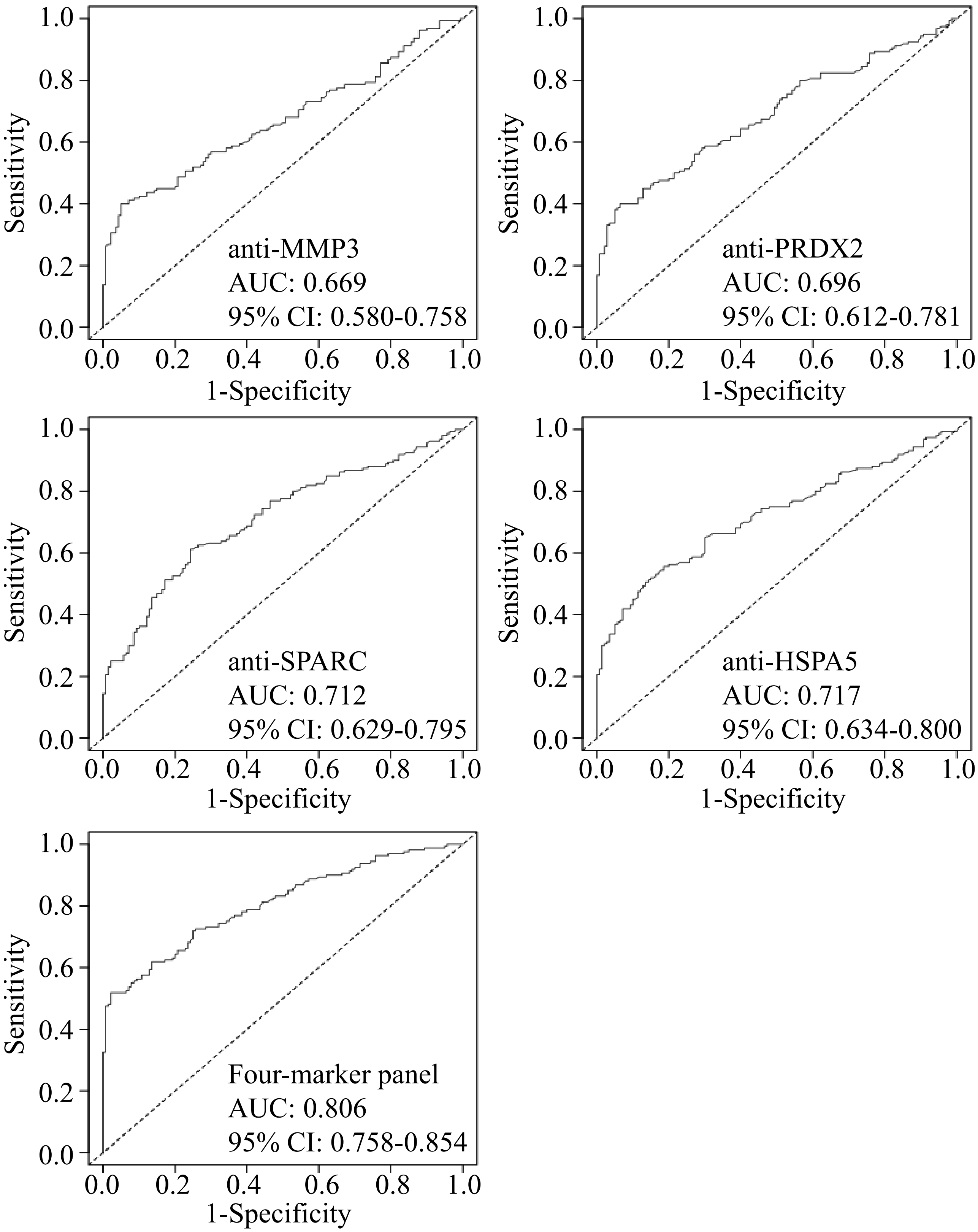

Supplementary Figure S3. Diagnostic efficacy of a marker panel consisting of anti-MMP3, anti-PRDX2, anti-SPARC, and anti-HSPA5. ROC curve analyses for the use of salivary anti-MMP3, anti-PRDX2, anti-SPARC, anti-HSPA5, and a marker panel consisting of the four auto-Abs in discriminating OSCC patients (*n* = 160) from controls (*n* = 140).

Supplementary Figure S4

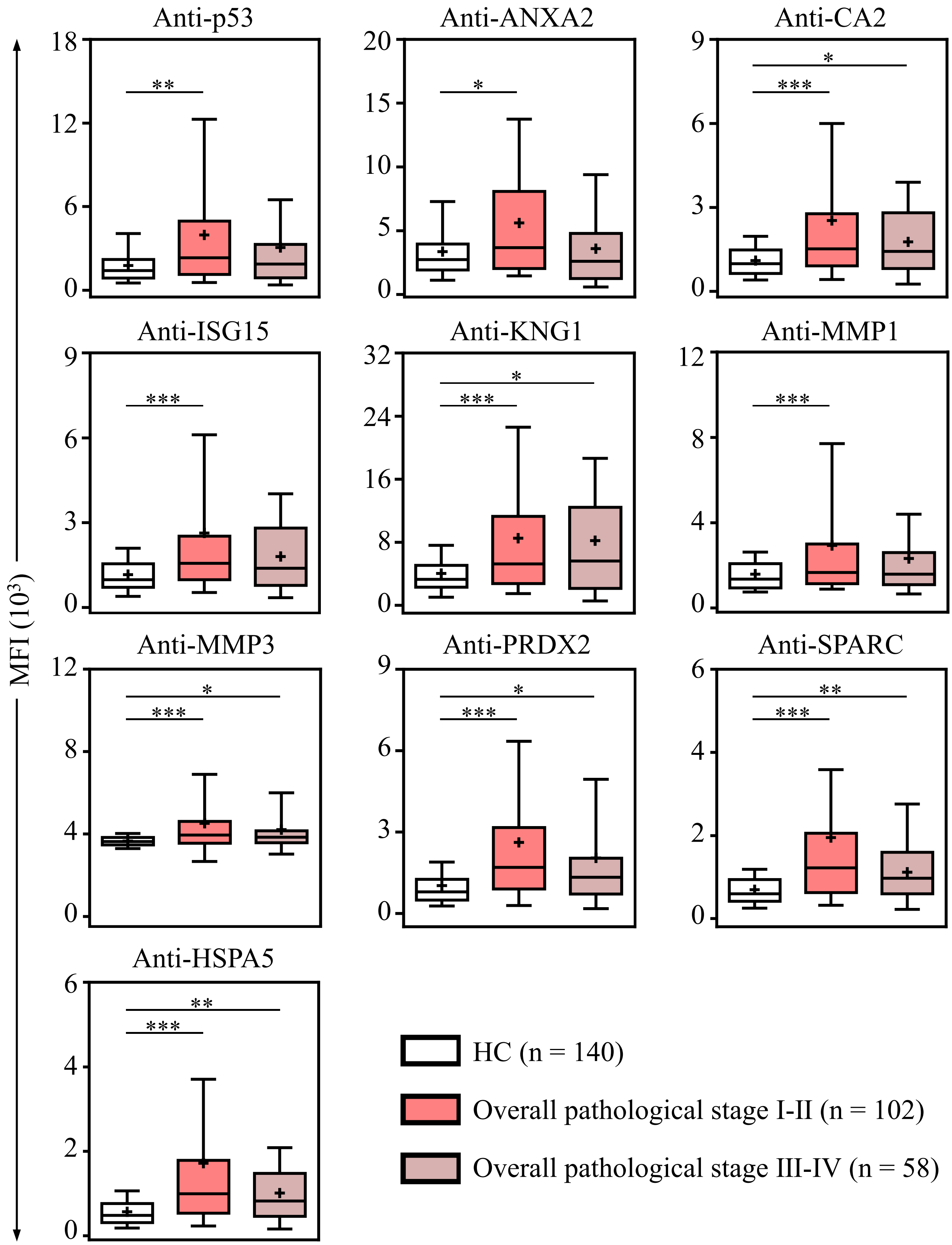

Supplemental Figure S4. Elevated levels of salivary auto-Abs in patients with OSCC at overall pathological stage I-II. The auto-Abs levels were measured in the salivary samples from the healthy controls (HC; n = 140), the patients with OSCC at stage I-II (n = 102) and III-IV (n = 58) using the multiplexed immunoassay. Data are presented as the upper and lower quartiles (box); the median value (horizontal lines), and the middle 90% distribution (whiskers) of median fluorescence intensity (MFI). *, $p < 0.05$, **, $p < 0.01$ and ***, $p < 0.001$.

Supplementary Figure S5

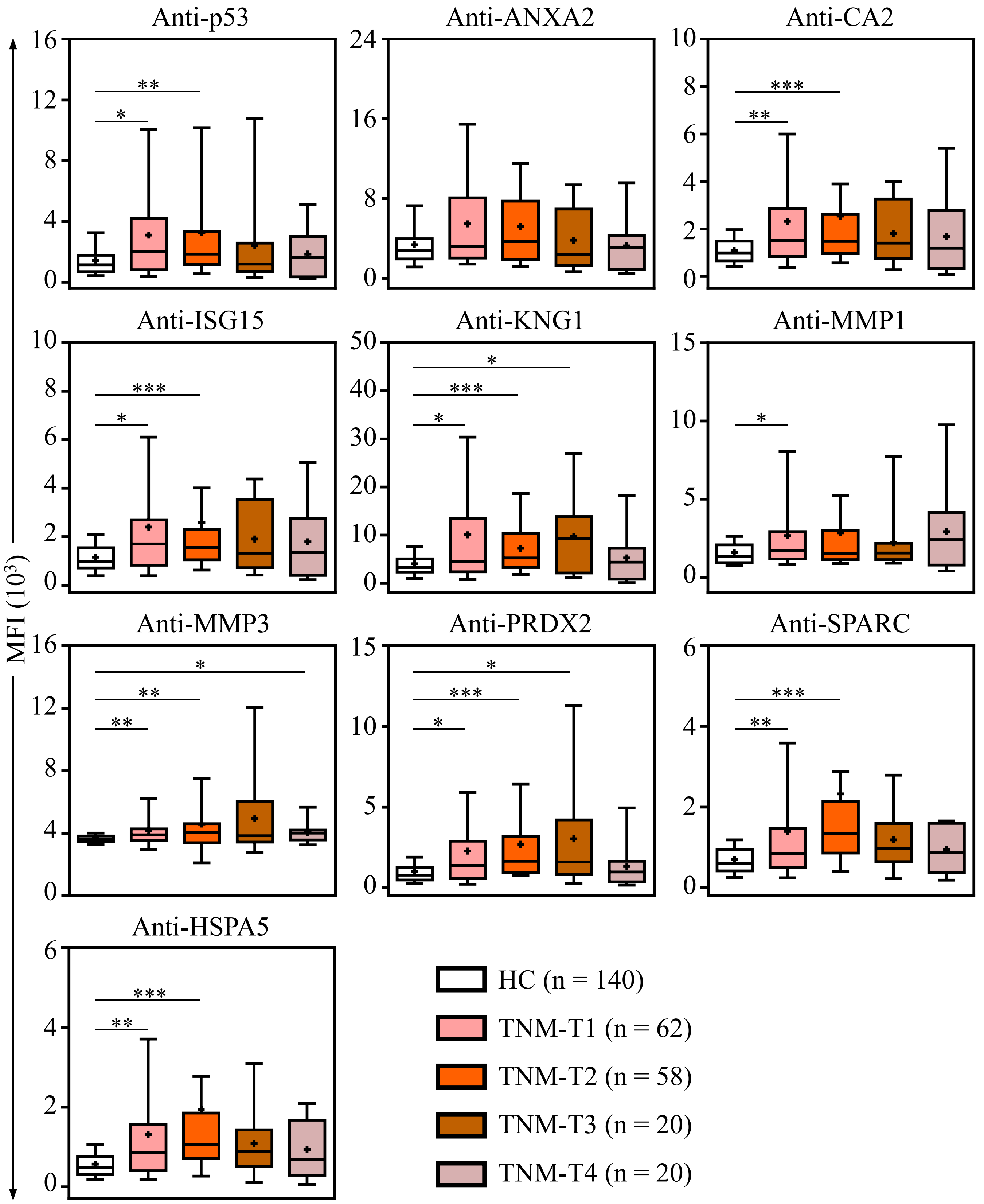

Supplemental Figure S5. Potentials of salivary auto-Abs as biomarkers for detection of early-stage OSCC. The auto-Abs levels were measured in the salivary samples from the healthy controls (HC; n = 140), the patients with OSCC at TNM-T status of T1 (n = 62), T2 (n = 58), T3 (n = 20), and T4 (n = 20) using the multiplexed immunoassay. Data are presented as the upper and lower quartiles (box); the median value (horizontal lines), and the middle 90% distribution (whiskers) of median fluorescence intensity (MFI). *, $p < 0.05$, **, $p < 0.01$ and ***, $p < 0.001$.

Supplementary Figure S6

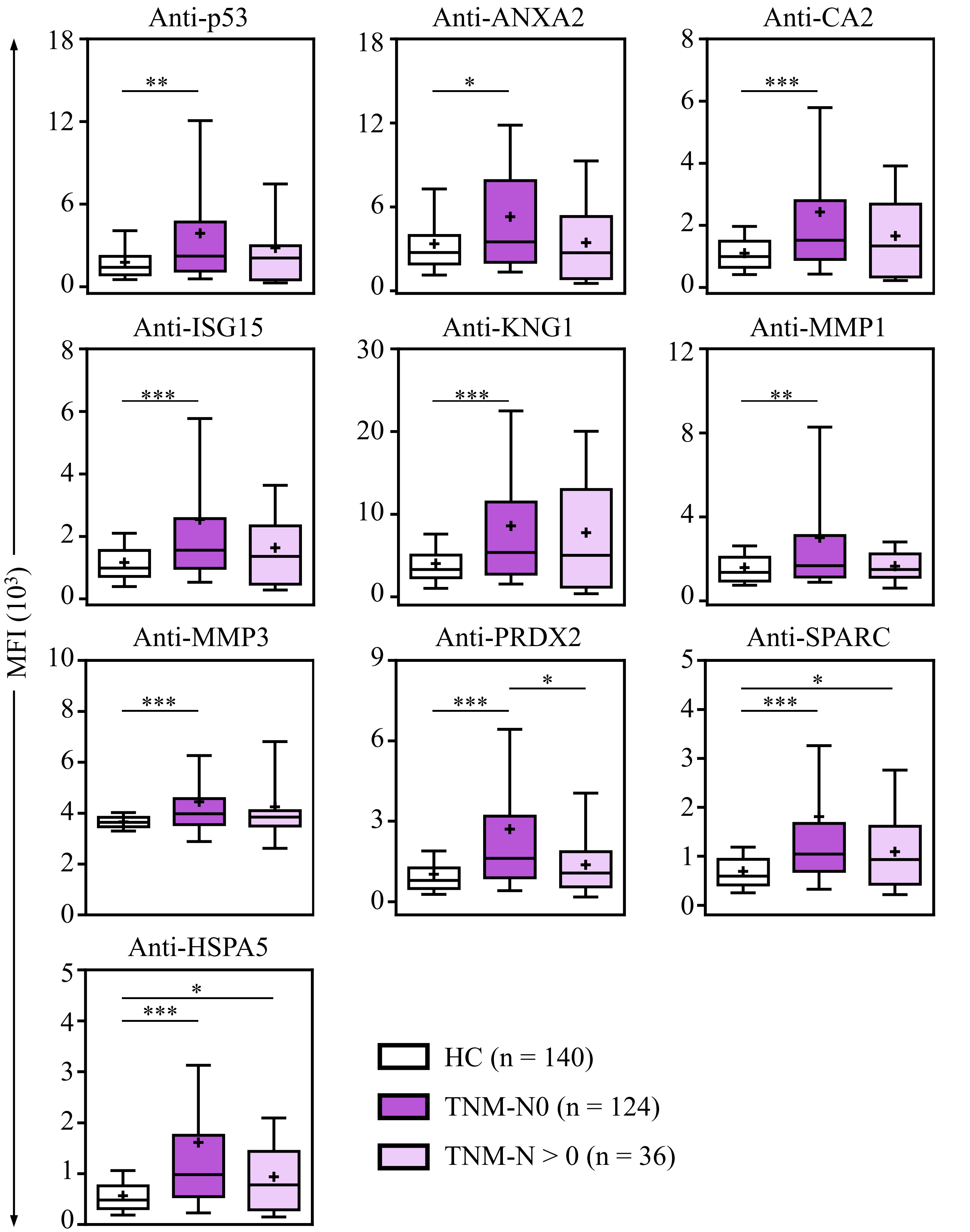

Supplemental Figure S6. Increased levels of salivary auto-Abs in patients with OSCC without lymphatic metastasis. The auto-Abs levels were measured in the salivary samples from the healthy controls (HC; n = 140), the OSCC patients without (TNM-N0; n = 124) and with (TNM-N1/N2; n = 36) lymphatic metastasis using the multiplexed immunoassay. Data are presented as the upper and lower quartiles (box); the median value (horizontal lines), and the middle 90% distribution (whiskers) of median fluorescence intensity (MFI). *, $p < 0.05$, **, $p < 0.01$ and ***, $p < 0.001$.

Supplementary Figure S7

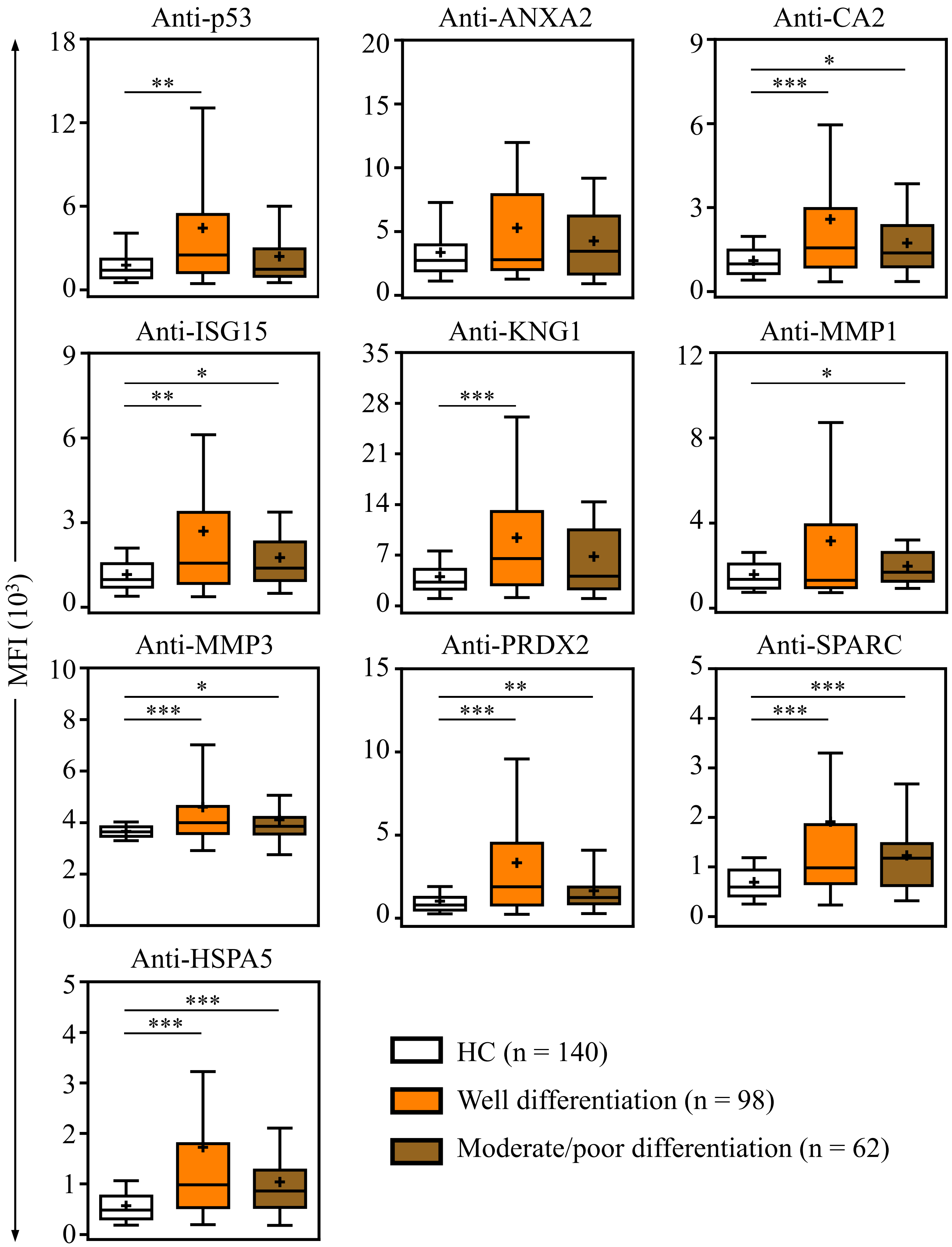

Supplemental Figure S7. Elevated salivary levels of auto-Abs in patients with well-differentiated OSCC. The levels of auto-Abs were detected in saliva samples from healthy controls (HC; n = 140), patients with well- (n = 98) and moderately/poorly (n = 62)-differentiated OSCC using the multiplexed bead-based system. Results are shown with median fluorescence intensity (MFI) of the level of auto-Ab and presented as the upper and lower quartiles (box), the median value (horizontal lines), and the middle 90% distribution (whiskers) of MFI. *, $p < 0.05$, **, $p < 0.01$ and ***, $p < 0.001$.
