# Supplementary material for: Development of a salivary autoantibody biomarker panel for diagnosis of oral cavity squamous cell carcinoma
Source: Front Oncol. 2022 Oct 31;12:968570. doi: 10.3389/fonc.2022.968570 (PMC9659860; doi:10.3389/fonc.2022.968570)
Supplement: Supplementary file 2 [file Table_1.pdf]

Supplementary Table S1. List of median of fluorescence intensity (MFI) acquired in the 10-plex immunoassay

Sample	Median of fluorescenec intensity (MFI)									
	anti-p53	anti-ANXA2	anti-CA2	anti-ISG15	anti-KNG1	anti-MMP1	anti-MMP3	anti-PRDX2	anti-SPARC	anti-HSPA5
1	682.0	2872.0	799.0	940.0	3083.0	444.0	2676.0	920.0	578.5	485.5
2	1364.0	2316.0	1323.0	1018.5	15355.0	1163.3	2502.0	1159.5	880.5	556.0
3	2396.0	1670.0	853.5	931.0	3998.5	1136.5	3403.0	1493.5	523.0	410.0
4	793.5	4099.0	1038.0	1013.0	2591.5	541.0	2172.0	808.0	819.5	475.0
5	1384.0	1937.5	1180.5	1018.0	7104.0	1813.8	3029.0	1890.5	826.5	988.0
6	1284.0	2360.0	430.0	380.0	1845.5	717.8	2231.0	730.5	240.0	204.5
7	2128.0	3090.0	655.0	757.0	5396.0	4591.0	3063.0	2319.0	408.0	841.0
8	2667.0	7826.0	718.5	884.0	6734.0	2147.8	3076.0	1076.0	361.0	273.5
9	2484.0	2963.5	1795.0	1847.0	3899.0	1374.8	2646.0	1490.5	995.0	765.0
10	729.5	1989.0	435.0	578.0	2413.5	2766.5	3470.5	1654.0	264.0	329.0
11	513.5	925.0	410.0	366.0	4022.0	578.0	2711.5	812.0	294.0	261.0
12	618.0	1397.0	648.0	881.0	3221.0	974.3	3760.5	2036.0	464.0	461.5
13	1356.0	2039.0	1842.0	1916.5	2693.5	645.5	1791.0	1287.5	1027.0	542.0
14	396.5	2890.5	206.0	285.0	798.0	378.0	2415.0	697.0	178.5	151.5
15	821.0	2705.0	617.5	820.0	2280.0	1072.0	8670.5	2117.0	432.0	492.0
16	207.0	225.0	203.0	233.0	714.0	258.3	2318.0	622.0	126.0	110.0
17	2677.0	8084.0	1183.0	1126.0	3446.0	4194.5	3173.0	1328.0	750.0	465.0
18	1240.0	2310.0	1349.5	1385.0	2114.0	1119.5	2388.5	1283.0	1025.5	823.0
19	3422.0	5337.0	2168.0	2172.0	5877.5	3826.0	3885.5	2345.0	1230.0	689.0
20	1639.0	12669.5	1459.0	1694.0	1782.0	1713.0	3324.5	2030.5	1213.5	935.5
21	1926.0	16396.0	1915.0	2403.0	2642.0	2146.0	4253.0	2131.0	1649.0	1009.0
22	1016.0	1950.0	926.0	946.0	2577.0	887.5	3396.5	1181.0	576.0	566.0
23	1589.0	2504.0	1042.5	2117.0	3825.0	8198.0	13379.5	5789.0	652.0	1722.0
24	417.5	839.0	220.0	592.0	321.0	551.3	6707.5	1577.5	152.0	198.0
25	2427.5	5561.5	1130.0	1465.0	3210.0	2337.5	4035.0	2408.0	773.0	873.0
26	2869.5	4516.0	2176.0	2350.0	7390.0	4450.8	3636.5	2150.0	1499.0	750.5
27	1285.0	3610.0	1191.0	1030.0	4273.0	1381.8	3783.0	1752.5	627.0	413.0
28	1488.0	2984.0	1693.5	1747.0	5313.0	2757.8	5285.5	2781.5	789.5	669.0
29	1338.0	1711.0	867.5	1129.0	12686.5	2986.3	2084.0	1363.5	501.0	934.0
30	4955.0	2766.0	1953.0	1919.5	3980.0	1816.8	5468.5	2458.5	1263.0	987.0
31	1460.0	2755.5	1683.0	1454.0	7716.0	3213.5	2823.0	1366.0	1058.0	565.5

32	1118.0	2257.0	792.0	807.5	3891.0	968.8	1926.0	1022.5	499.5	283.0
33	1182.0	1745.0	1119.5	951.0	5080.5	1017.5	2443.5	1435.0	579.5	463.5
34	814.5	2823.0	692.0	721.0	2389.0	1552.3	2013.5	1530.5	450.5	380.0
35	3930.0	8264.0	3480.0	3711.5	3885.5	2087.8	3883.5	2902.0	2714.0	1655.0
36	1923.0	5712.0	1075.0	1300.0	3672.0	1167.8	4207.0	1616.0	839.0	623.5
37	1318.5	2714.0	2019.0	2122.0	1031.0	1152.5	4040.5	2562.0	1952.0	1408.5
38	1656.0	2426.0	1066.0	935.0	2781.0	1197.3	1824.0	889.0	626.0	312.5
39	1363.5	2130.0	1751.0	2035.5	1317.0	983.3	3965.5	1182.5	1595.0	672.0
40	1804.0	2705.5	1148.5	1353.0	7132.0	1714.0	4007.0	1464.0	754.0	507.5
41	2027.0	3921.0	1512.0	1648.5	2799.0	1225.3	2185.0	842.5	1504.5	515.0
42	1719.0	1994.5	421.5	559.0	478.5	908.0	2508.5	1041.0	221.0	257.0
43	998.5	1695.0	799.0	1047.0	2335.0	1784.8	2251.5	1058.0	521.5	402.0
44	770.5	2261.0	745.0	821.5	2466.0	476.8	2600.0	1139.0	511.0	376.5
45	1199.0	1107.0	420.0	1032.0	2690.5	2181.0	5416.0	2679.5	287.0	953.0
46	2462.0	2998.0	2018.0	1727.0	3019.0	1958.0	3213.5	1563.5	1288.0	534.5
47	2176.0	1110.0	509.5	544.0	3566.0	413.5	1513.5	752.5	344.0	233.0
48	1492.0	3460.0	999.0	1493.0	3522.5	2206.0	3613.0	2293.0	538.0	784.5
49	2856.0	3348.0	999.0	838.5	1539.5	2312.8	3670.0	2143.0	538.0	451.5
50	929.5	4483.0	582.0	856.0	2272.5	1755.0	2909.5	1487.5	376.0	360.0
51	1606.5	4974.5	602.5	645.5	3221.0	1600.5	2836.0	1428.5	376.0	341.0
52	7333.0	4431.0	3208.0	3195.0	5338.0	3757.3	11193.0	5866.0	1748.5	2806.0
53	902.0	2746.0	908.5	924.5	2019.0	884.3	2900.5	1327.0	698.0	542.0
54	486.0	3029.0	249.0	238.0	1040.0	1056.8	1075.0	504.0	166.0	114.5
55	1772.0	3912.5	1479.0	1687.0	8731.0	1191.8	4021.0	2479.5	1061.0	906.0
56	4321.0	3185.0	1067.0	913.0	6134.0	1935.5	2602.0	1416.5	701.5	612.5
57	1598.5	1926.0	852.0	777.0	5039.0	1014.8	1274.0	883.0	557.0	336.0
58	757.0	3593.5	738.0	575.0	1968.5	424.5	1733.5	713.0	471.0	333.0
59	4191.5	1914.0	1278.0	1141.0	13674.0	4030.5	2162.5	910.0	872.0	432.0
60	866.0	1379.0	805.5	614.0	2126.0	991.0	2167.0	849.0	578.5	398.0
61	4037.0	3311.5	990.5	780.0	9605.0	1539.0	3302.0	1076.0	532.0	355.0
62	449.0	486.0	349.0	349.5	645.0	316.8	2019.0	750.5	246.0	195.0
63	4215.0	8149.0	1486.0	1321.5	4229.5	2619.8	5479.0	2882.0	853.0	1012.5
64	4103.0	4123.5	2212.0	1740.0	10035.0	3731.0	5041.0	2142.0	1647.0	1057.0
65	1516.5	2276.0	1354.5	1103.0	4320.0	1203.0	1617.0	782.5	972.5	356.5

66	698.5	2665.0	618.0	659.5	3627.5	580.5	2978.0	1000.0	446.0	443.0
67	704.0	765.0	714.0	651.0	3347.0	744.8	2791.5	1088.0	458.0	395.0
68	1330.5	3965.0	788.0	797.5	2824.0	906.0	2113.0	808.0	571.0	354.0
69	1419.0	7471.0	657.0	503.0	2350.0	3510.5	2742.0	1660.0	382.0	332.5
70	372.0	454.0	394.5	294.5	701.5	248.3	1511.0	366.0	223.0	117.5
71	2315.0	1963.0	1487.0	1374.5	5234.0	904.8	2651.5	1012.5	1068.0	518.5
72	747.0	1754.0	548.0	728.0	2456.5	3508.0	3026.5	1348.0	345.0	291.0
73	878.0	867.0	645.0	568.0	3463.0	392.3	2205.0	948.0	387.0	418.0
74	629.0	1949.5	355.0	434.0	604.5	2265.0	1702.0	628.5	289.5	166.0
75	1157.5	1840.0	1110.0	1159.0	4548.5	1280.5	2737.0	1801.0	853.0	707.0
76	1060.0	2878.5	658.0	679.5	2533.0	1922.3	1404.0	825.0	487.0	279.5
77	3636.0	4230.5	1550.0	1365.5	10950.0	1851.5	1591.5	1019.5	999.0	415.5
78	3235.0	4915.0	2195.0	2118.0	3917.0	880.0	4107.0	1350.0	1308.5	596.0
79	1823.0	2697.0	915.5	955.0	4786.0	1314.0	2147.5	984.0	519.0	686.5
80	1236.0	1753.5	1118.0	1140.0	6538.0	935.5	1288.0	714.0	955.0	419.0
81	1197.0	2095.0	937.5	879.0	1314.0	517.3	1779.0	801.0	702.0	345.5
82	1338.5	2128.0	1159.0	1181.5	5470.5	2094.8	2577.0	1274.5	866.0	563.5
83	1803.0	5121.5	942.0	1005.5	3447.5	2387.5	2731.0	1075.0	650.0	346.0
84	5973.0	10998.0	3703.0	3313.0	10367.0	2232.5	4701.0	2439.0	2678.0	1237.0
85	851.0	1946.0	1109.0	1490.0	2283.0	690.5	2607.5	1324.0	1102.0	1007.0
86	2180.5	2878.0	1549.0	1597.5	2354.5	1168.0	1591.5	1008.0	1316.5	739.0
87	4550.5	4789.0	2580.0	2329.5	4997.0	2595.3	3118.0	1721.0	1807.5	820.5
88	3022.0	4174.0	2013.0	1926.0	9239.5	1118.3	1791.0	1090.0	1585.0	730.5
89	416.0	605.0	155.0	178.0	228.0	159.5	1175.5	231.0	115.0	80.0
90	2855.5	4745.0	2579.0	2487.0	5073.0	1608.5	3468.5	1784.0	1677.0	774.5
91	2504.0	5893.5	1126.0	1674.0	6477.5	3072.0	3490.0	1837.0	1004.5	821.0
92	591.5	1686.5	771.0	756.5	3681.0	1123.5	2235.0	973.0	551.5	448.5
93	4965.5	9549.5	3971.0	3845.0	14515.5	5114.5	5164.0	3302.0	2332.5	1081.5
94	471.5	744.0	429.0	433.0	678.5	353.0	2653.0	933.5	223.0	233.0
95	2020.5	7156.0	1973.0	1907.0	6278.0	1921.8	4283.0	1770.5	1234.0	714.5
96	136.0	233.5	42.0	93.5	102.0	108.3	2195.0	431.0	25.0	60.0
97	3553.0	1589.0	2047.0	2082.0	4684.5	3017.8	2414.5	1366.0	1514.0	800.5
98	1045.0	879.0	826.5	527.0	2808.0	1236.8	2660.0	1070.0	295.0	323.5
99	1519.0	1466.0	903.0	941.0	1388.0	518.5	3322.0	1146.5	594.0	342.5

100	2717.0	1771.0	2537.0	2067.0	4660.0	1545.3	2140.5	1202.5	1882.0	702.5
101	3775.5	1731.5	420.0	360.5	1930.0	1529.5	1278.0	585.0	277.0	137.5
102	936.5	3362.0	789.0	637.0	1994.5	1392.8	2216.5	1292.5	429.0	490.0
103	1116.0	1602.0	627.5	718.0	1474.0	681.3	2611.0	1026.5	552.0	425.0
104	1980.5	3003.0	1584.0	2220.5	16220.0	1389.0	3844.5	2196.5	1505.0	1190.5
105	292.0	515.0	331.0	323.5	905.0	835.0	2189.0	583.0	226.0	162.0
106	1677.0	2102.0	1525.5	1446.0	6659.5	1261.3	2719.0	1328.5	1198.5	653.5
107	2376.0	2579.0	1139.0	1106.0	1739.0	793.0	1731.0	681.0	922.0	478.5
108	1009.0	4002.0	807.5	753.0	4516.5	789.5	1504.5	815.0	626.0	439.0
109	6312.0	7452.0	3033.0	3203.5	7076.0	2473.5	6980.5	2752.0	2476.0	1751.5
110	1458.5	2187.5	1618.0	1216.0	9986.0	1257.5	2929.0	1488.0	968.0	658.5
111	1412.0	2158.0	1549.0	1424.0	27651.5	2593.3	2655.0	1406.5	1277.0	783.0
112	995.5	6081.0	1224.5	2191.0	2082.0	1388.3	8775.5	1226.0	1034.5	671.0
113	2309.0	3750.0	2970.0	3214.0	3531.0	1630.5	2379.0	2174.5	2263.0	971.0
114	868.0	3122.5	1008.0	1048.0	3441.5	1882.3	3296.0	1818.5	662.0	723.0
115	1311.0	3980.0	1725.0	1413.0	5525.5	1491.8	3688.5	1767.0	837.5	522.0
116	2004.0	1779.0	1806.0	1705.5	3197.0	3245.0	2910.0	1673.0	1296.0	893.0
117	2675.0	2870.5	1415.0	1313.0	5406.0	1862.5	2126.5	1255.5	959.5	425.5
118	1973.5	3124.0	1761.0	2106.0	3548.5	5491.8	2855.0	1712.0	1750.0	1056.0
119	864.0	1815.0	939.0	834.5	2353.0	543.0	1455.5	790.5	519.0	328.5
120	2843.5	19549.0	2551.0	2374.0	25745.0	4272.5	5830.0	1803.0	1822.0	750.5
121	1009.0	4783.0	904.0	826.0	4947.0	563.8	1396.0	647.5	645.5	493.0
122	2100.0	2797.0	1295.0	1220.0	2990.0	842.5	2370.0	1363.0	745.0	457.0
123	1968.0	2287.0	2213.5	1735.5	4807.0	503.5	2218.0	949.0	1386.0	549.0
124	782.0	1237.5	306.0	312.0	1029.0	463.5	2237.0	672.5	188.0	147.5
125	1464.0	2065.0	955.0	881.0	17169.5	1107.3	2153.5	1150.0	545.0	455.0
126	472.0	566.5	219.5	257.0	1606.0	1055.3	841.0	573.0	206.0	127.5
127	379.0	864.0	311.0	383.0	353.5	393.5	1694.5	628.5	258.5	285.0
128	1295.0	2469.0	574.0	782.0	2292.5	2240.8	9733.5	2238.0	324.0	501.0
129	2554.0	9072.5	3653.5	3571.0	2790.0	2460.8	5096.0	4200.5	3273.5	2913.0
130	612.5	3194.0	805.5	607.0	2781.0	745.0	4013.0	1006.5	410.0	424.0
131	567.0	1086.0	473.0	497.5	2532.0	399.0	2685.0	707.5	243.0	209.0
132	833.0	1811.0	928.0	1001.0	4811.0	731.8	2941.0	1131.5	791.0	536.0
133	1038.0	2031.0	503.5	597.0	1190.5	652.8	1900.0	1002.5	378.5	310.5

134	414.5	1757.0	214.0	246.0	966.0	471.5	2287.0	559.0	152.0	118.0
135	501.0	1165.0	450.5	313.5	2508.0	392.3	1287.0	655.5	244.0	246.0
136	3295.0	2083.0	616.0	595.0	2447.5	496.5	1243.5	818.5	473.5	269.5
137	630.0	706.0	342.0	358.5	2713.0	364.0	2974.0	755.0	223.0	247.5
138	945.0	2028.0	759.0	835.0	599.0	608.3	1433.0	891.5	641.0	469.5
139	2207.0	3061.0	1918.0	2006.0	6261.0	1123.0	5375.5	1631.5	874.5	612.0
140	2036.5	2749.5	1277.0	1180.0	8834.0	987.8	1939.0	1065.5	968.0	453.0
141	2654.0	5905.5	2001.0	2695.0	9882.0	4903.8	5876.5	3198.0	1456.0	1119.0
142	1848.0	2121.0	967.5	1063.0	2791.0	1216.5	3087.0	1815.5	654.5	714.0
143	1526.5	3671.5	895.0	1532.0	9435.0	1837.3	8918.0	2998.0	561.0	1008.5
144	1756.5	1842.0	928.0	1458.0	9399.5	1827.5	4548.0	2533.5	787.0	782.5
145	4182.0	3913.0	2390.0	2104.0	13541.5	1954.5	3187.0	1836.0	1687.0	850.0
146	2065.0	2440.0	1208.0	1131.5	3315.0	1074.0	2009.5	1360.0	898.0	641.0
147	611.0	503.0	263.5	298.0	2157.5	465.5	1362.0	461.5	171.0	127.0
148	1577.0	4612.0	1543.0	1511.0	4877.0	3067.8	1050.5	1974.0	889.0	39.0
149	1511.0	980.0	1572.0	1536.0	16201.0	720.3	1596.0	752.5	1127.0	415.5
150	1206.5	4670.0	1004.5	1382.0	2879.0	2278.5	3168.0	1359.5	653.0	564.0
151	6761.5	5514.5	5306.0	4893.0	25064.5	2615.5	5289.0	2467.5	3544.0	1149.0
152	1189.5	1568.0	1483.0	1032.0	8324.0	514.8	2002.0	1214.5	718.0	580.0
153	452.0	810.0	342.0	429.0	1480.5	476.8	1951.5	669.5	198.5	186.0
154	2190.0	2994.5	1842.0	1665.0	6348.5	4129.8	5127.5	2204.0	1019.0	811.0
155	1692.0	1969.5	1133.5	1144.5	3739.5	1566.3	2823.0	1962.0	752.0	835.0
156	421.5	1301.5	291.0	352.5	796.0	2082.0	2797.5	680.5	253.0	275.5
157	1664.0	1210.0	1455.0	1474.0	13558.0	672.5	3361.0	1598.5	1175.5	776.0
158	774.0	3254.0	675.0	842.5	4346.0	2286.5	2568.0	1057.0	773.0	501.0
159	1261.5	2180.5	756.0	711.0	2070.0	656.5	2913.0	604.5	438.0	312.0
160	796.0	1018.5	725.0	719.0	2081.0	1273.8	1962.0	796.5	537.0	408.0
161	1003.0	595.5	1308.5	1045.0	2798.0	287.5	2066.0	910.0	778.5	358.0
162	2147.0	5323.0	3910.0	3100.0	13420.5	1892.5	4784.0	2544.0	2270.0	1778.0
163	591.5	1482.5	1006.0	859.0	2536.5	457.5	2443.5	1562.0	532.0	622.5
164	7527.5	4325.0	3284.5	2568.0	9411.5	8693.5	6634.5	5232.0	1785.5	1966.0
165	773.0	799.5	755.0	674.0	3332.0	416.5	1744.0	857.0	493.0	496.0
166	584.0	534.0	491.0	445.0	3361.0	282.5	1798.5	787.0	289.5	264.5
167	4451.5	2193.0	3629.5	3468.5	10738.0	768.3	3502.0	1994.0	3495.5	1176.5

168	882.5	2294.0	513.0	437.5	1976.0	949.3	2201.5	503.0	358.0	218.0
169	1937.0	3098.5	1300.0	1404.0	9438.5	1222.0	2010.0	1202.0	994.0	524.5
170	1384.0	1790.0	1410.0	1392.5	6929.0	904.8	3579.0	1460.5	1007.5	694.5
171	3716.5	2708.0	2150.5	2575.0	3806.5	1437.5	5796.0	2700.0	2008.0	1325.5
172	4206.0	4272.5	3665.0	3818.0	6589.0	2511.8	3620.5	2355.5	3160.0	1703.5
173	5801.5	4677.5	5155.5	4811.0	12128.0	3976.0	6432.0	3193.0	4172.5	2382.0
174	2921.0	7707.5	1994.0	2534.0	2500.0	3104.5	4826.5	1922.5	1779.0	933.0
175	1366.0	3776.0	1515.0	2139.0	2151.0	1377.0	3127.0	1736.5	1628.0	960.0
176	1467.0	1907.5	1179.0	1154.0	4810.0	500.0	1298.0	934.0	913.0	466.0
177	2839.0	4235.0	2973.0	3379.0	5622.0	2297.3	2362.0	1091.0	2067.0	765.0
178	2483.5	2034.5	1447.0	1476.0	6814.0	1092.0	1833.0	1242.5	1180.0	482.5
179	1764.5	2059.0	1759.0	1743.0	3407.0	1349.5	1965.0	1375.5	1303.5	747.0
180	3392.0	6174.0	1988.0	1780.5	7422.5	2052.0	3501.5	1340.0	1251.0	494.0
181	1284.0	1416.0	826.0	939.0	4909.0	732.5	2614.0	1445.5	678.5	590.0
182	2634.0	2679.0	1827.5	1800.0	5519.0	1086.0	2677.0	1499.0	1325.5	732.0
183	10782.5	4334.0	4105.0	4043.0	3332.0	1867.5	3609.5	2097.5	3373.5	1702.5
184	1603.5	1922.5	1547.5	1199.0	9407.5	1238.0	4181.0	1230.5	719.0	566.0
185	869.5	1824.5	734.0	853.5	3987.5	871.0	1777.0	1180.0	529.0	346.0
186	7351.0	7307.0	5710.0	5325.0	12761.0	8159.3	5705.0	3915.0	4568.0	2310.5
187	3961.0	4533.0	2641.5	2405.0	22372.0	1483.0	2617.0	1435.0	1846.0	696.0
188	10951.5	13162.0	4489.0	6469.0	17486.0	15535.3	18744.0	10533.0	2481.0	2602.5
189	6057.5	7135.0	4031.5	4294.0	8163.0	5179.5	7073.5	2567.5	2741.5	1488.0
190	4367.5	6288.5	2115.5	2739.0	17702.0	4442.5	5898.5	3673.0	1262.5	946.0
191	4837.0	9163.5	2208.0	3788.0	3192.5	5531.0	10921.0	5446.5	1644.0	1250.0
192	7467.5	4456.0	1309.0	1902.0	17202.0	3123.8	5353.5	2577.5	960.0	911.0
193	363.0	455.5	203.5	283.0	895.5	242.3	2525.0	590.0	155.0	130.5
194	3034.0	3231.5	2444.0	3200.5	2258.0	2782.3	6612.0	2851.0	2120.0	1159.5
195	2316.0	2428.0	1274.0	1240.5	13235.0	2260.0	3467.5	1274.5	749.0	484.0
196	255.0	888.0	311.0	354.0	553.0	360.8	1753.5	615.0	226.0	175.0
197	2520.5	1162.5	1459.0	1402.0	2636.5	1142.3	2445.0	852.5	958.5	571.0
198	2958.0	4232.0	1902.0	1988.5	6962.0	1310.5	2619.0	1368.5	1201.0	588.0
199	1027.5	1285.5	870.0	811.0	9879.5	675.3	1587.0	881.0	551.0	262.5
200	3963.0	3339.0	3864.0	3417.0	5498.0	754.3	4675.5	1995.0	2437.0	727.0
201	770.0	2220.5	400.0	356.0	2489.0	478.0	1576.0	515.5	262.0	163.0

202	2586.0	3048.0	2341.0	2033.0	31667.0	3092.0	2836.0	2206.0	1047.0	516.0
203	2508.5	4798.0	1504.0	1252.5	6723.5	630.8	2777.0	1212.0	958.5	529.0
204	1466.0	2817.0	823.0	834.0	6368.0	779.0	2652.0	1239.0	578.0	572.5
205	2312.0	3760.0	2126.0	1833.0	6364.0	1450.3	2051.5	873.0	1420.0	491.5
206	13364.0	8997.0	5959.0	6124.0	22148.5	4739.0	5956.5	3517.0	4694.0	1736.0
207	7167.0	7865.0	3076.0	3225.0	10996.0	6759.8	5911.0	2699.5	1897.0	1137.0
208	7888.0	14273.0	3838.0	3635.0	26609.0	4173.3	5385.5	2529.0	2290.5	991.0
209	2928.0	16257.5	1976.0	2334.0	6830.0	2297.8	6658.5	2934.5	1199.5	792.0
210	412.0	2027.0	319.5	377.0	576.0	377.8	2145.0	675.5	209.0	185.0
211	16100.0	7370.0	3865.0	3572.0	18161.0	4231.8	7360.5	4302.0	2314.0	1147.0
212	2598.0	3525.5	2167.0	2542.5	2923.0	1193.5	1600.0	886.0	1233.0	576.0
213	738.0	2054.0	777.5	861.0	2514.0	658.3	2281.0	826.5	706.5	400.5
214	1774.0	4504.0	1500.5	1351.0	7245.0	1492.8	2339.5	1275.0	1005.5	575.5
215	1989.5	2042.0	1035.0	1157.0	4374.0	1044.3	1541.0	903.0	980.0	493.0
216	3056.0	12051.0	1638.0	2215.5	5370.5	4436.3	8894.0	3679.0	903.0	872.0
217	1391.0	9227.0	1370.0	1741.0	8184.0	2223.0	3499.5	1632.0	873.0	518.0
218	2115.5	8640.0	1812.5	1921.0	9881.0	1075.0	1961.5	1111.5	1577.0	717.0
219	2298.0	3491.5	1185.0	1322.5	1780.0	1329.8	2756.0	1201.5	805.0	445.5
220	1123.0	1824.0	1013.5	991.0	4395.0	942.8	2727.0	1145.0	579.0	361.5
221	1979.5	3558.0	2578.0	2839.0	7094.5	4405.0	4028.5	1543.5	1760.0	973.0
222	2549.0	2432.5	1974.5	1829.0	2405.0	1975.5	2421.5	1563.0	1531.5	799.0
223	511.0	1468.0	381.5	489.0	627.5	757.0	1310.0	887.5	259.0	378.0
224	1454.0	2631.0	926.0	1020.0	5528.0	1629.8	1710.0	1107.5	590.0	298.5
225	1103.5	1827.0	1070.0	1059.5	6213.5	702.8	1391.0	714.5	876.0	448.0
226	4239.5	2565.0	5528.5	5011.0	7709.0	2787.0	7828.0	3963.5	3150.5	1714.5
227	4657.0	2505.0	2506.5	2176.0	5308.0	1984.3	2950.5	1638.5	1480.0	826.5
228	1150.0	1634.0	1361.5	1406.0	4141.5	814.8	2513.0	1316.5	1008.0	722.0
229	3742.5	4806.0	2333.0	1964.0	5146.0	2723.5	5871.5	2346.0	1212.5	780.5
230	2426.0	2080.5	1679.0	1245.0	8656.5	622.8	1650.0	1054.0	1246.5	679.0
231	1502.0	1376.0	964.0	749.0	4449.0	425.8	2276.5	1173.0	504.5	411.0
232	1033.0	1124.5	1042.5	818.0	3824.0	718.0	1477.0	802.0	639.0	363.5
233	6559.5	10158.0	3734.0	3256.0	12926.0	5137.0	4293.0	2336.0	2168.0	955.5
234	692.0	3765.0	439.0	619.5	2368.0	1525.3	2458.0	1139.0	306.5	241.0
235	2997.0	6099.0	4101.0	4380.0	14754.0	1864.8	4948.0	2547.5	3455.0	1309.0

236	5010.0	6050.0	2034.0	1780.0	12639.5	3567.5	5772.5	2753.0	1099.5	846.0
237	394.0	717.0	861.0	536.5	1393.5	569.5	3350.5	1075.5	326.0	212.5
238	1887.0	1649.0	876.0	882.0	10605.5	975.8	1708.0	1195.0	618.0	400.0
239	1563.0	2270.0	1468.0	1189.0	2480.0	2622.0	2706.0	1149.0	822.5	411.5
240	25421.5	24651.0	21962.0	23005.5	18924.5	15825.8	27753.5	20979.5	14707.0	13904.0
241	12821.0	11288.0	6596.0	6386.0	21894.0	10783.5	10872.5	8036.0	3793.0	3399.0
242	14920.0	15490.0	12086.5	12894.0	32508.0	15602.3	13570.5	10647.0	7343.0	4369.0
243	1352.0	2727.0	1479.0	1906.0	4134.0	1883.8	6302.0	2780.0	1008.0	912.0
244	5645.0	7545.0	3758.0	3976.0	17901.0	4498.5	7211.5	5087.0	2580.5	1658.0
245	13186.0	11285.5	6098.5	6056.5	31449.0	10942.3	9703.5	5314.0	3965.0	2135.0
246	10679.0	17360.0	6367.0	7043.0	22201.0	12055.5	15326.0	8484.0	4174.0	2972.5
247	6419.0	9399.0	2938.0	3949.0	6747.5	9703.0	12541.0	9500.0	2227.5	2386.5
248	440.5	1465.5	154.0	197.5	1986.0	371.0	1887.5	854.0	106.0	141.0
249	1977.0	2424.0	1790.5	1702.0	13685.0	1378.5	2961.0	1620.0	1483.0	913.0
250	1631.0	2390.5	908.0	904.0	4745.0	857.5	1564.5	1249.5	658.5	423.5
251	3133.5	6948.0	2801.0	2694.5	13537.0	2149.8	4332.0	2985.0	1870.5	1551.0
252	14349.0	9421.5	3008.5	4009.0	12273.5	6295.8	17571.0	8700.0	1833.0	3453.0
253	8413.0	9957.0	3485.5	3906.0	26509.0	12142.8	15274.5	9768.0	2240.0	3559.0
254	1451.0	1239.0	1260.0	1555.5	2766.0	1392.8	6361.0	3423.5	918.0	758.0
255	2941.0	9227.0	2736.0	2396.5	4172.0	5508.0	7343.0	4994.0	1443.0	1520.0
256	2928.5	3804.0	2703.0	2328.0	18581.0	3286.5	5096.0	2714.0	1455.0	1341.0
257	447.5	1232.0	348.5	411.0	1181.5	480.3	2870.0	1056.0	150.5	192.5
258	3077.0	13471.0	1937.0	2461.0	7900.5	3847.8	13487.5	4736.5	1099.0	1413.5
259	1412.0	3684.0	1433.5	1278.5	4809.0	2422.0	3855.0	1326.5	852.5	572.0
260	2468.0	1897.0	1305.0	1563.0	2900.0	4684.5	5478.5	2638.0	712.5	643.0
261	6016.0	8114.0	3557.5	2475.0	8712.5	4077.3	4762.0	3230.5	1589.0	1137.5
262	1541.0	5025.5	1733.0	1725.5	3826.5	2863.3	2663.0	1671.5	1238.5	881.5
263	3557.0	4579.5	869.0	1542.0	4756.0	2149.5	6841.0	4551.0	809.0	1857.0
264	961.5	4257.0	898.0	1255.0	2255.0	2307.3	3274.0	1828.0	582.5	540.0
265	944.0	1111.0	874.5	943.0	1322.5	376.0	3434.0	1586.5	600.0	745.0
266	5166.0	2053.0	422.5	527.5	4478.5	513.0	3300.0	1295.5	325.0	328.0
267	1130.0	3508.5	1472.0	1178.0	4159.0	795.5	6913.5	3142.5	688.0	1380.0
268	496.0	866.0	334.0	432.0	988.0	848.8	5209.0	2348.0	233.0	404.0
269	600.0	3822.0	555.0	568.0	6894.0	863.3	4063.5	1598.0	238.5	363.0

270	267.0	467.5	75.5	228.0	150.0	307.3	3972.5	422.0	51.0	83.0
271	500.0	533.0	234.0	286.0	422.0	332.0	2287.0	686.5	128.0	139.0
272	537.5	1851.0	733.0	1040.0	635.0	1019.8	3987.0	1793.5	372.5	766.5
273	1357.0	3808.0	911.0	1359.0	2812.0	1886.3	4859.0	2267.0	590.5	583.0
274	380.0	590.5	259.5	476.0	1268.0	560.8	5317.5	1770.0	203.0	273.0
275	2633.0	16113.0	1366.5	1954.0	6644.0	3402.0	5939.0	3294.5	905.5	794.0
276	536.0	893.0	268.0	713.0	981.0	1272.3	1634.5	1356.0	189.5	312.5
277	472.0	675.0	186.0	470.0	4645.0	685.0	4875.0	1748.0	136.0	372.0
278	1597.5	4205.0	1340.0	1703.0	5613.0	2037.5	3437.0	2067.0	1175.5	707.0
279	431.5	497.0	593.5	656.5	827.0	334.5	1946.0	582.0	322.0	148.0
280	1116.5	869.0	491.0	738.5	961.0	826.5	2248.0	1682.0	335.0	418.5
281	1737.5	2576.0	1315.0	1536.0	11404.5	2235.5	3815.5	2103.0	887.0	924.0
282	1046.0	4646.0	1836.0	2217.0	1305.0	1492.0	4613.0	2388.5	1529.0	1169.0
283	1633.5	4467.5	868.0	883.5	1204.0	1741.5	3243.0	1609.0	428.0	341.5
284	2158.5	10561.0	1507.5	2032.0	3087.5	3295.5	7820.0	3137.0	1058.0	1233.0
285	6558.5	9502.0	3480.0	4419.0	4762.0	12908.8	12528.5	6070.0	1936.0	1659.0
286	445.0	446.0	311.0	490.0	423.0	472.0	3078.5	902.0	218.0	227.0
287	2091.0	2775.0	1901.5	2279.5	7763.0	2619.5	4349.5	2312.5	1611.0	1281.5
288	463.5	275.0	419.0	528.0	748.5	270.5	1705.5	583.0	305.0	191.0
289	9979.0	9769.5	4322.0	5091.0	13413.5	6002.3	9829.5	5393.5	2943.0	2117.0
290	2921.0	4300.0	2607.0	2451.0	19101.5	1903.8	2737.0	1630.0	1914.0	854.0
291	21030.0	10323.5	5019.5	5011.0	32725.5	11211.3	6901.5	3717.0	3493.5	1931.0
292	1366.0	2461.5	926.0	1174.0	5010.0	2351.5	3201.0	1737.5	658.5	515.0
293	2811.0	2741.5	1842.0	2900.0	7261.0	4208.5	6988.0	3327.5	1167.0	1058.5
294	4452.0	4135.0	2438.0	2663.0	32724.0	4604.5	4139.0	2397.0	2071.0	1239.5
295	1424.5	2872.0	994.0	1054.5	3008.0	1110.5	2570.5	1352.0	709.0	581.0
296	1446.5	2389.5	1053.0	1090.0	3671.0	565.0	2230.0	1148.5	950.0	775.5
297	658.5	631.5	531.5	474.0	5521.5	402.5	2075.0	806.0	377.5	224.0
298	12230.5	8474.0	4622.0	4734.0	7743.0	7021.8	5830.0	3051.5	3082.0	1624.0
299	1722.0	2566.0	1667.0	1423.0	7945.0	715.5	2196.0	1330.0	965.0	442.0
300	845.5	963.0	716.0	710.0	1621.5	451.5	5269.0	3439.0	505.5	955.0
