# Supplementary material for: Development of a salivary autoantibody biomarker panel for diagnosis of oral cavity squamous cell carcinoma
Source: Front Oncol. 2022 Oct 31;12:968570. doi: 10.3389/fonc.2022.968570 (PMC9659860; doi:10.3389/fonc.2022.968570)
Supplement: Supplementary file 3 [file Table_2.pdf]

Supplementary Table S2. ROC curve analysis of auto-antibody (auto-Ab) markers

| Auto-Ab | OSCC ($n$ = 160) | | OSCC at overall pathological stage I-II ($n$ = 102) | |
|---|---|---|---|---|
| | Sig. | AUC (95% CI)[a] | Sig. | AUC (95% CI)[a] |
| Anti-p53 | 0.009 | 0.624 (0.535-0.714) | 0.006 | 0.647 (0.544-0.751) |
| Anti-ANXA2 | 0.138 | 0.570 (0.479-0.662) | 0.036 | 0.612 (0.507-0.716) |
| Anti-CA2 | < 0.001 | 0.666 (0.579-0.753) | < 0.001 | 0.688 (0.589-0.787) |
| Anti-ISG15 | < 0.001 | 0.661 (0.574-0.748) | < 0.001 | 0.687 (0.588-0.786) |
| Anti-KNG1 | 0.001 | 0.661 (0.574-0.749) | 0.001 | 0.677 (0.577-0.777) |
| Anti-MMP1 | 0.001 | 0.615 (0.525-0.704) | 0.015 | 0.630 (0.527-0.733) |
| Anti-MMP3 | 0.016 | 0.669 (0.580-0.758) | 0.001 | 0.674 (0.566-0.782) |
| Anti-PRDX2 | < 0.001 | 0.696 (0.612-0.781) | < 0.001 | 0.725 (0.628-0.822) |
| Anti-SPARC | < 0.001 | 0.712 (0.629-0.795) | < 0.001 | 0.732 (0.635-0.828) |
| Anti-HSPA5 | < 0.001 | 0.717 (0.634-0.800) | < 0.001 | 0.741 (0.646-0.836) |

[a] Receiver operating characteristic (ROC) curve is used to evaluate the ability of atuo-Abs for discriminating OSCC from healthy individual group. Data are shown as area under ROC curve (AUC) values with 95% confidence interval (CI).
